# Dramatic nucleolar dispersion in the salivary gland of *Schwenkfeldina* sp. (Diptera: Sciaridae)

**DOI:** 10.1038/s41598-021-87012-5

**Published:** 2021-04-16

**Authors:** José Mariano Amabis, Eduardo Gorab

**Affiliations:** Departamento de Genética e Biologia Evolutiva, Instituto de Biociências, Universidade de São Paulo, Rua do Matão 277, Cidade Universitária, São Paulo, SP 05508-090 Brazil

**Keywords:** Chromosomes, Cell biology, Molecular biology

## Abstract

Micronucleoli are among the structures composing the peculiar scenario of the nucleolus in salivary gland nuclei of dipterans representative of Sciaridae. Micronucleolar bodies contain ribosomal DNA and RNA, are transcriptionally active and may appear free in the nucleoplasm or associated with specific chromosome regions in salivary gland nuclei. This report deals with an extreme case of nucleolar fragmentation/dispersion detected in the salivary gland of *Schwenkfeldina* sp. Such a phenomenon in this species was found to be restricted to cell types undergoing polyteny and seems to be differentially controlled according to the cell type. Furthermore, transcriptional activity was detected in virtually all the micronucleolar bodies generated in the salivary gland. The relative proportion of the rDNA in polytene and diploid tissues showed that rDNA under-replication did not occur in polytene nuclei suggesting that the nucleolar and concomitant rDNA dispersion in *Schwenkfeldina* sp. may reflect a previously hypothesised process in order to counterbalance the rDNA loss due to the under-replication. The chromosomal distribution of epigenetic markers for the heterochromatin agreed with early cytological observations in this species suggesting that heterochromatin is spread throughout the chromosome length of *Schwenkfeldina* sp. A comparison made with results from another sciarid species argues for a role played by the heterochromatin in the establishment of the rDNA topology in polytene nuclei of Sciaridae.

## Introduction

Transcriptional activity of ribosomal RNA (rRNA) genes in specific chromosome regions is the primary event for the local assembly of the nucleolus, the starting site of ribosome biogenesis. Light microscopy enables to observe such a nuclear entity in most cells but the optimal, detailed nucleolar visualisation under the same optical device is found in salivary gland cells of dipterans, one of the cell types where giant, polytene chromosomes develop. These chromosomes, that are an exceptional case of the cell cycle, are formed from chromosomes of diploid nuclei by duplication rounds of chromatids. At the end of each S-phase, DNA strands do not segregate, remaining paired and parallel to each other. Cell division does no occur as polytene cells do not enter mitosis, so that they remain in interphase. Among other features shared by polytene chromosomes, DNA under-replication is of particular interest in relation to this report. Such a phenomenon is usually found in heterochromatic regions and results from partial sequence replication as a possible consequence of stalled replication forks. Stalled forks are structurally unstable and are apt to collapse leading to DNA breakage and loss^1^.

Among Diptera, Sub-Order Nematocera, representatives of Sciaridae constitute a morphologically peculiar and unusual example of nucleolus as observed in salivary gland nuclei of species within this family.

Early findings in several sciarid species showed the nucleolus at the pericentric end of the X chromosome in polytene cells^2–6^. However, the sciarid nucleolus tends to fragment throughout the larval development so that, in contrast to well formed nucleoli from dipterans such as *Drosophila*^7^ and chironomid species^8^, a prominent nucleolus is not seen in the salivary gland nuclei of Sciaridae. Another type of nucleolar material named micronucleoli also occurs in the sciarid salivary gland. These structures containing DNA and RNA appear as small, round bodies scattered in the nucleoplasm and can be associated with certain chromosome regions^5,9–11^.

The development of the in situ hybridisation method at the end of the decade of 1960 allowed the identification of chromosomal regions containing rRNA genes, or ribosomal DNA (rDNA), and produced the first results in sciarid species. In *Sciara coprophila*, radioactively labelled rRNA probes hybridised to the heterochromatic end of chromosome X and to micronucleoli^12–15^. In *Rhynchosciara hollaenderi,* heterochromatic ends of chromosomes X and C hybridised consistently to rRNA. In this species, hybridisation to specific regions of chromosomes A and B appeared to be dependent on the presence of micronucleoli attached to those chromosomal sites^12^*.* Early data in *Rhynchosciara americana* chromosomes^16^ showed the rDNA localisation similar to its closely related species, *R. hollaenderi*.

Later, non-isotopic in situ hybridisation applied to *R. americana* showed in addition an rDNA site at the non-centromeric end of the X chromosome^17–19^. In *Rhynchosciara baschanti* rRNA genes are mainly located in the proximal end of the X chromosome. However, evidence for rDNA sites in the three polytene autosomes of the latter species was also presented^20^. In *Trichomegalosphys pubescens* (formerly named *Trichosia pubescens*), the rDNA is localised in the heterochromatin of the X chromosome and in free micronucleoli^18,21^ namely the same pattern described previously for *S. coprophila*. In relation to all sciarid flies studied with nucleolar focus, a very short account on the rDNA localisation in *Schwenkfeldina* sp. showed an unusually high number of rDNA hybridisation sites associated to the salivary gland chromosomes. In contrast, non-polytene chromosomes display the nucleolar organiser region (NOR) in a single locus, a feature shared by other sciarid species^18^.

While research on sciarid NORs has long been initiated, structural peculiarities restricted to this dipteran family have remained obscure. In particular, multiple NOR sites detected by rDNA probes in the salivary gland, in contrast to a single location in mitotic chromosomes. Also, variable chromosomal locations of NORs in the salivary gland of sciarid species that have been studied, some of which closely related, are intriguing and deserved a more detailed investigation in this work. *Schwenkfeldina* sp. was chosen to be studied in this report because of its unique, unexpected degree of nucleolar fragmentation and dispersion, detected by in situ hybridisation, compared to any other sciarid species that have been studied with a nucleolar focus. We thought that this extreme case of nucleolar dispersion could help to identify genomic features or even some functional link implied in the exceptional nucleolar pattern in terms of localisation in the *Schwenkfeldina* salivary gland, which was described shortly in a previous work^18^.

In order to better understand such an unusual picture in this species, chromosomal localisation of the rDNA in distinct cell types, its relative proportion in polyene and diploid cells as well as epigenetic markers were assessed in this fly. Also, a comparison of *Schwenkfeldina* results with those from another sciarid fly, *Rhynchosciara americana*, take part in this work. The data suggest that rDNA dispersion is dependent on polytenisation and chromosomal localisation of the heterochromatin in the species analysed. Support for rDNA dispersion as a device to restore the rDNA loss due to under-replication in *Schwenkfeldina* is also provided.

## Results

### Early cytological observations on *Schwenkfeldina* salivary gland chromosomes

**Figure 1:**
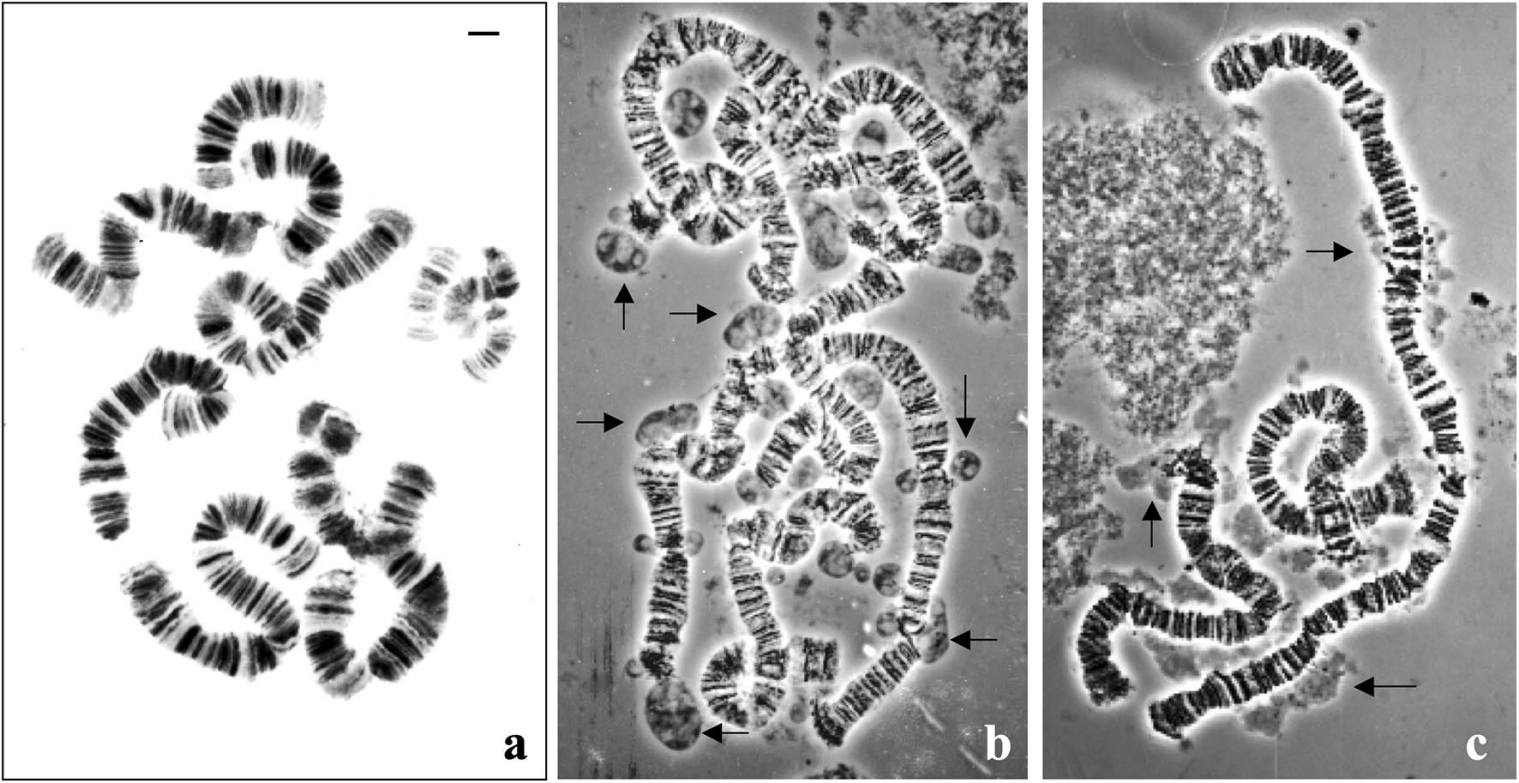
(**a–c**) Polytene chromosomes of *Schwenkfeldina* sp. (**a**) Orcein staining photographed under bright field allows chromosome visualisation but not extrachromosomal structures of interest to this work. (**b**,**c**) Phase contrast images showing two morphological aspects often observed in the nuclear entities of this species. The arrows point to some examples of structures, either disconnected or attached to the chromosomes, that were eventually identified as micronucleoli on the basis of in situ hybridisation results. (**b**) Nucleolar bodies displaying sharp edges and exhibiting some internal structure and (**c**) micronucleoli with less sharp edges and apparently devoid of internal structure. Bar = 15 µm.

Polytene chromosomes of *Schwenkfeldina* sp. commonly display several breakpoints that sometimes make difficult a rapid identification of its basic chromosome number (Fig. 1a). However, the frequency of connections between chromosome fragments as well as observations on mitotic chromosomes showed that this species has a basic chromosome number of four (Fig. 1a, Fig. S1, Fig. S3, Fig. S11). In contrast to the other sciarid species that have been studied, nucleolar-like material in the *Schwenkfeldina* salivary gland is not primarily associated with one particular chromosome (Fig. 1b,c). As *Schwenkfeldina* sp. larval groups used for slide preparation reached the adult stage, the nucleolar dispersion is not a consequence of abnormal larval development. Micronucleolar-like bodies appeared associated with several regions of the four polytene chromosomes and sometimes were also dispersed in the nucleoplasm, apparently disconnected from the chromosomes. Their morphology was not the same as that observed in *Sciara*, *Rhynchosciara* and *Trichomegalosphys* salivary glands in the sense that they were larger than those of the latter species. In addition, nucleolar bodies of *Schwenkfeldina* sp. sometimes display an apparent internal structure (Fig. 1b) while other situations show instead amorphous appearance (Fig. 1c). After examining fourteen slides, it was observed that the association of micronucleoli-like structures with chromosomal regions does not occur by chance. Chromosome termini, breakpoints and certain interstitial regions of a given chromosome appear recurrently associated with nucleolar-like bodies (Fig. S1).

### rDNA localisation features in the salivary gland of *Schwenkfeldina*

**Figure 2:**
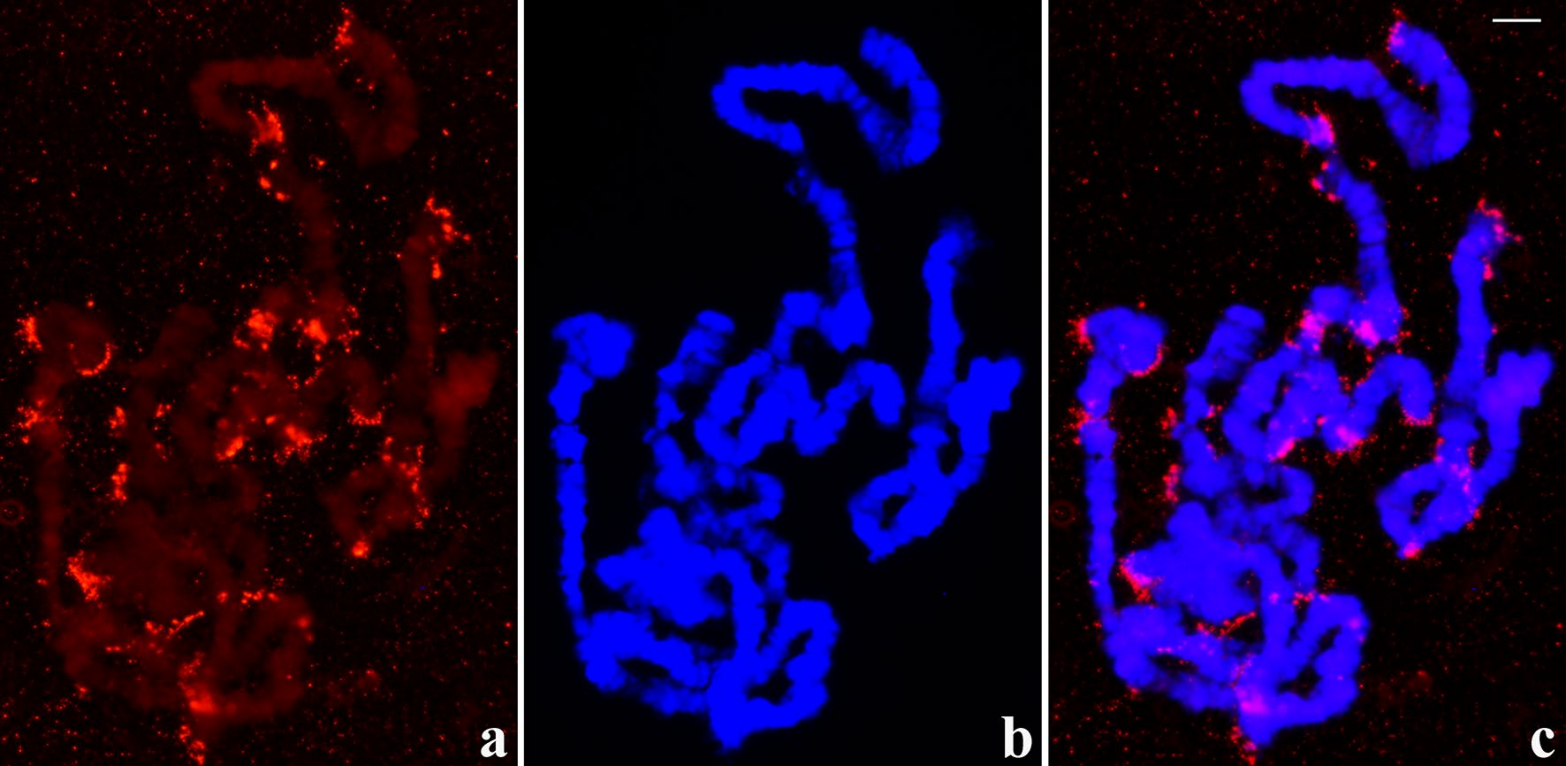
(**a–c**) Localisation of the rDNA in *Schwenkfeldina* sp. polytene chromosomes of the salivary gland showing significant rDNA dispersion as visualised by RNA probe signals (**a**), its corresponding DAPI staining image (**b**) and the merged signals (**c**) either disconnected or attached to the chromosomes. Bar = 45 µm.

As already observed in several sciarid species, the salivary gland is a long organ and winds up the body of the larva. Salivary gland cells placed in its anterior part undergo more DNA replication cycles than those composing posterior regions of the gland. Consequently, polytene chromosomes from the anterior part attain the highest polyteny degree. In situ hybridisation applied to these chromosomes usually shows significant rDNA dispersion. The most frequent pattern consists of rDNA probe signals at chromosome ends as well as attached to several interstitial sites, mostly chromosome breakpoints. Usually, no chromosomal site displaying a stronger hybridisation signal than other sites is detected, namely no main NOR was found (Fig. 2). In much less frequent cases, hybridisation signals expand to larger areas when compared to usual situations suggesting decondensation of the structures containing rDNA that are attached to chromosome regions (Fig. S2). The results of in situ hybridisation were reproduced many times and made possible a compilation of the most frequent chromosomal regions to which micronucleoli is attached (Fig. S3).

**Figure 3:**
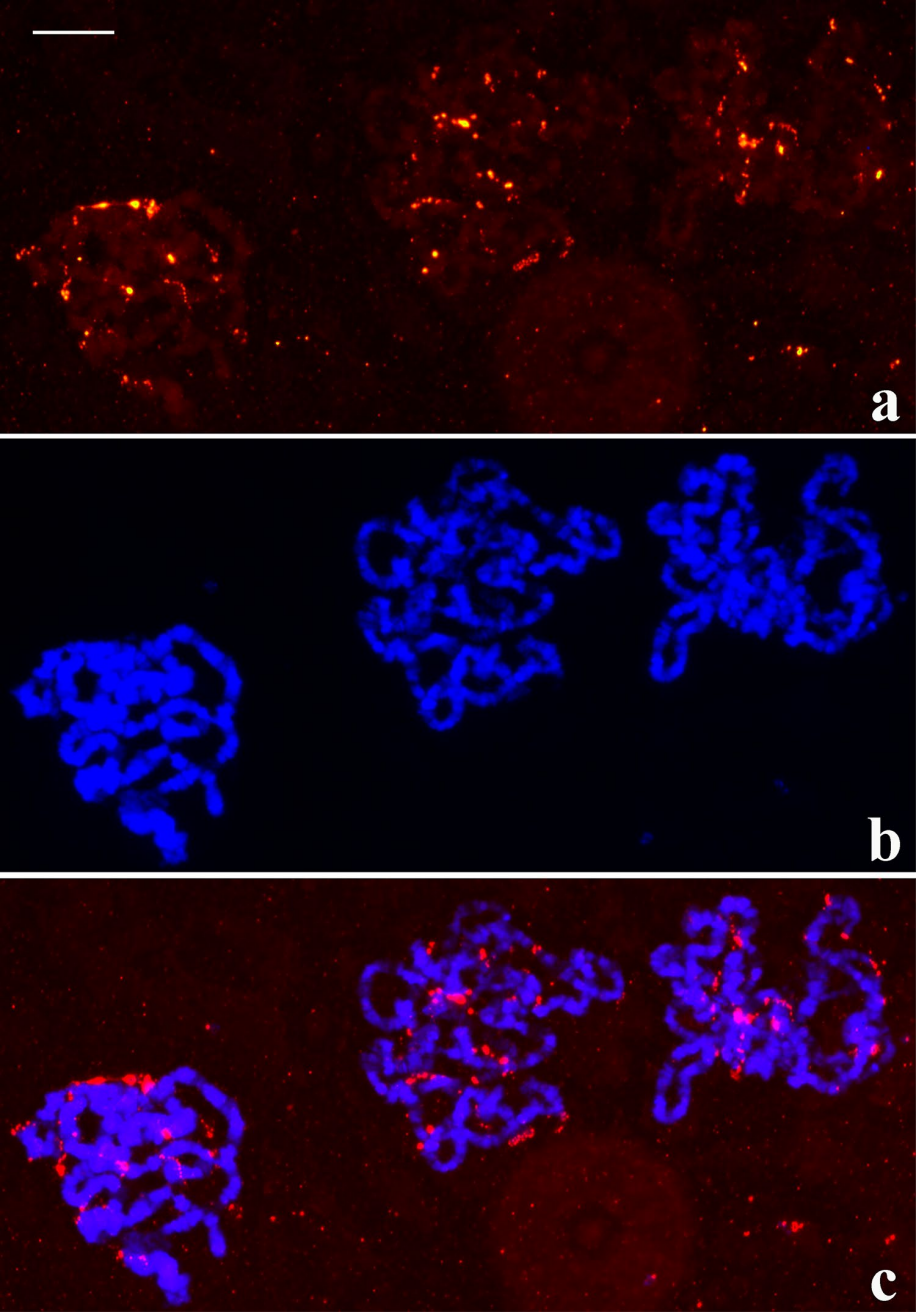
(**a–c**) Localisation of the rDNA in *Schwenkfeldina* sp. polytene chromosomes from posterior cells of the salivary gland showing rDNA dispersion at a lesser extent than nuclei from anterior cells as inferred by RNA probe signals (**a**), its corresponding DAPI staining image (**b**) and the merged signals (**c**). Bar = 65 µm.

Regarding sciarid salivary glands, they are usually composed of three cell types (S1, S2 and S3) that are grouped into sections throughout the salivary gland length. Anterior cells (S1) always exhibit chromosomes that have the highest polyteny degree. Chromosomes in posterior cells of the salivary gland (S2 and S3) display variable, lower polyteny degree and this provided additional information on the nucleolar fragmentation in *Schwenkfeldina* sp. For example, some polytene chromosomes whose polyteny degree is comparatively high (S2) but still lower than those from the anterior cells (S1) may exhibit significant rDNA dispersion so that a principal (NOR) is not visualised (Fig. 3). In contrast, polytene chromosomes from posterior cells showing very low polyteny degree (S3) enabled the identification of a main NOR site thanks to the intensity of its hybridisation signal. In addition, signs of rDNA dispersion probably from the main rDNA region were also detected (Fig. S4).

### Transcriptional activity of nucleolar bodies

**Figure 4:**
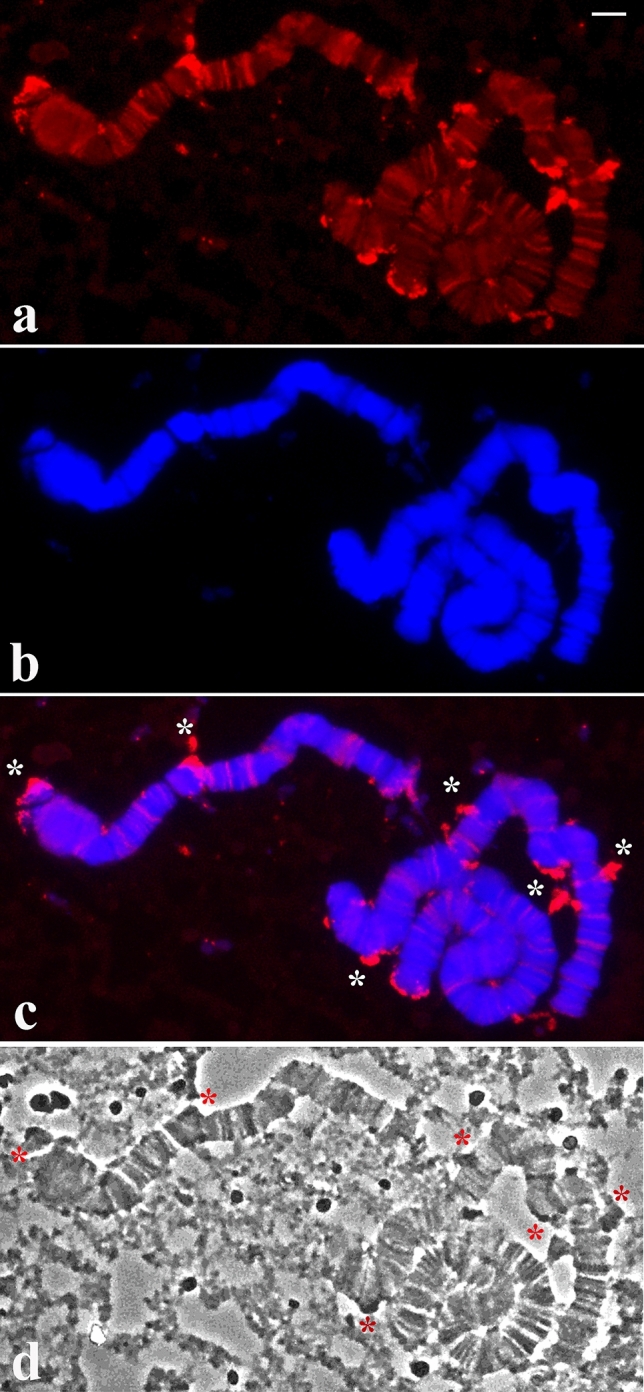
(**a–d**) Transcriptional activity in *Schwenkfeldina* sp. polytene chromosomes as visualised by the detection of endogenous RNA.DNA hybrids. Fluorescent antibody signals (**a**), the corresponding DAPI image (**b**), the merged signals (**c**) and the same chromosomes under phase contrast (**d**) The *asterisks* point to some examples in which fluorescent detection of endogenous hybrids is seen in micronucleolar bodies either disconnected or attached to the chromosomes. Bar = 15 µm.

The localisation of the rDNA in polytene chromosomes of this species raised the question whether ribosomal gene transcription is also spread in the nucleus or restricted to a micronucleoli subset. To address this issue, the procedure for the detection of endogenous RNA.DNA hybrids was used, namely the formation in situ of hybrid molecules composed of template DNA and the product of its own transcriptional activity^22–24^. Although, as for in situ hybridisation, use of this technique may lead to a considerable loss of nucleolar morphology, RNA.DNA hybrids were detected on entire regions coinciding with the presence of micronucleoli (Fig. 4). The reproduction of the method showed hybrid detection in the whole micronucleolar set and, in some slides, the hybrid signal occupied partially the micronucleolar area (Fig. S5). Controls performed with slides previously treated with RNAse A or RNase A/H showed no chromosomal labelling after completion of DNA.RNA hybrid detection method.

### rDNA localisation in Malpighian Tubules, neuroblasts and embryonic nuclei

**Figure 5:**
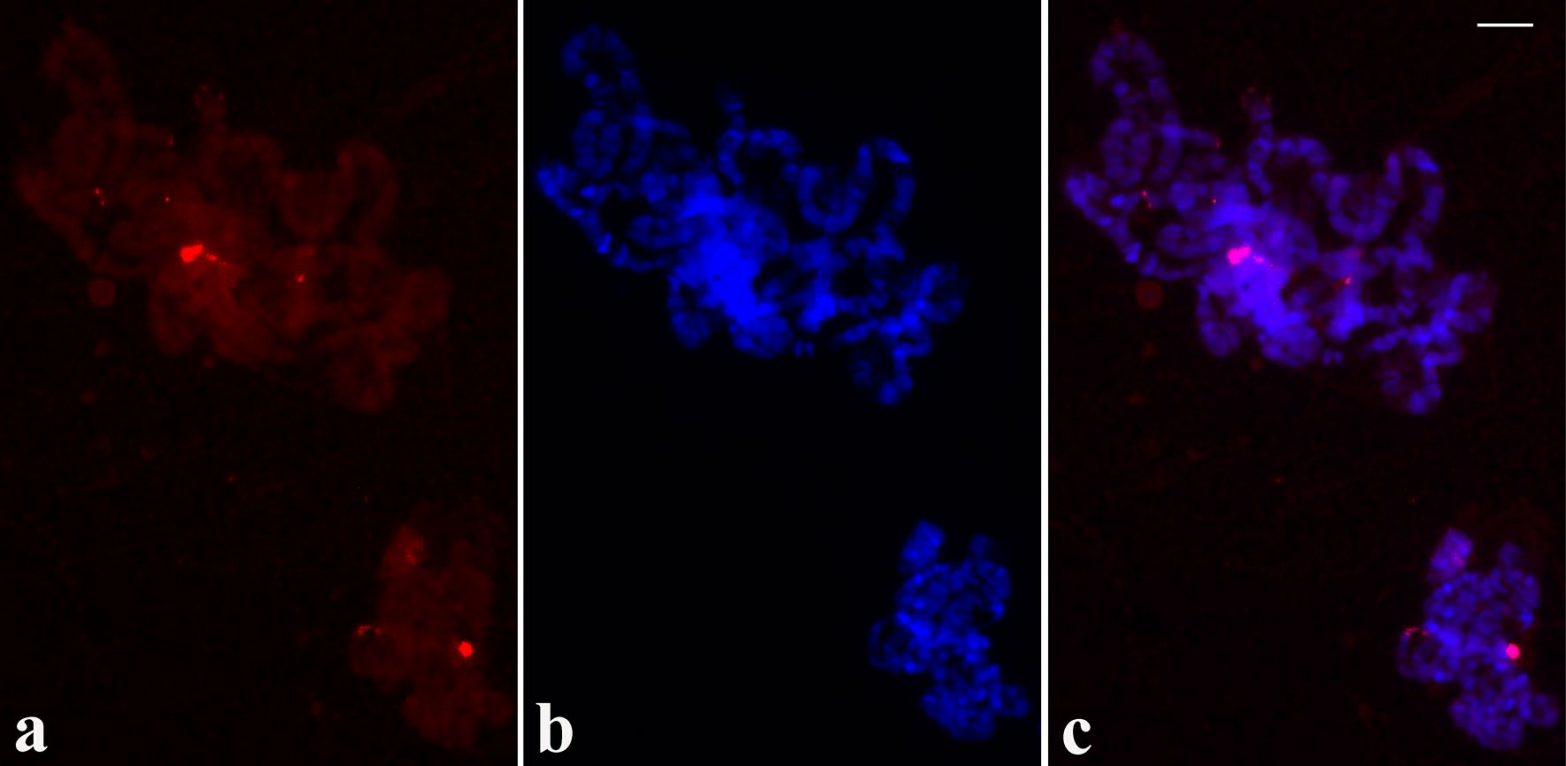
(**a–c**) Localisation of the rDNA in *Schwenkfeldina* sp. polytene chromosomes from Malpighian Tubules showing RNA probe signal (**a**), its corresponding DAPI staining image (**b**) and the merged signals (**c**). Dispersion of the rDNA in these nuclei is usually lower than that observed in posterior cells of the salivary gland. Bar = 25 µm.

**Figure 6:**
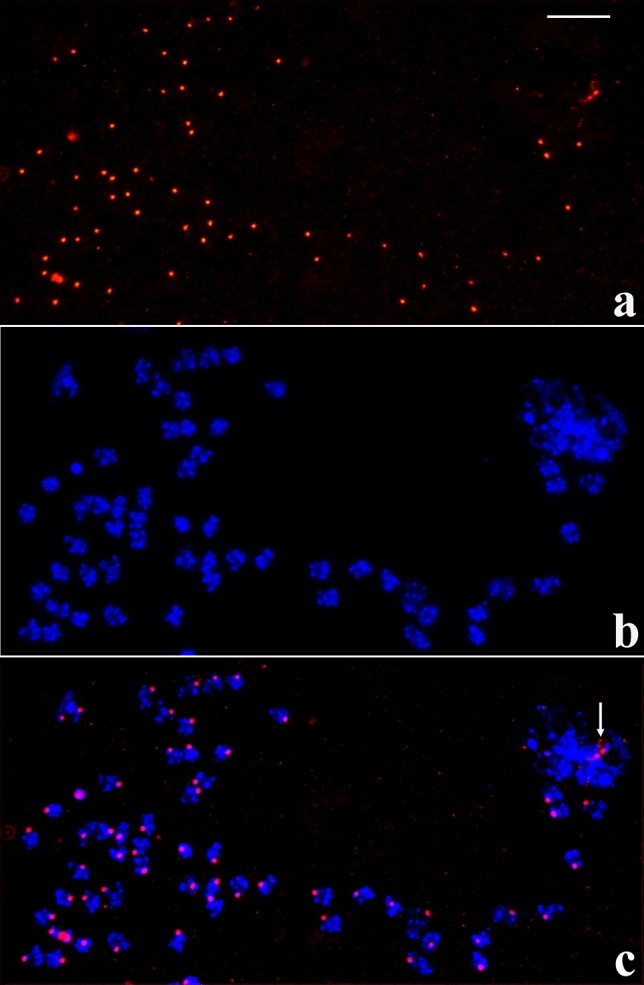
(**a–c**) Localisation of the rDNA in *Schwenkfeldina* sp. neuroblast nuclei showing RNA probe signals (**a**), its corresponding DAPI staining image (**b**) and the merged signals (**c**). Usually, a single rDNA signal is detected in diploid nuclei. The arrow points to a polytene chromosome set showing a low rDNA dispersion degree. Bar = 50 µm.

Usually, low polyteny is a feature of chromosomes in Malpighian Tubules of Diptera. In situ hybridisation results from several slides showed invariably a main probe signal and in some cases rDNA dispersion visualised as fluorescent spots either close to the main signal or farther than the apparent dispersion centre (Fig. 5). Among distinct cell types that are present in neuroblasts, diploid nuclei displayed a single probe signal. It probably represents signals from the somatic pairing of two alleles (Fig. 6). Similar results were obtained in early (1–3 days) embryonic nuclei of *Schwenkfeldina* sp. (Fig. S6). It is worth mentioning hybridisation results observed in certain neuroblast nuclei that underwent polyteny. These chromosomes display, in addition to the main signal, signs of rDNA dispersion (Fig. 6) such as those detected in Malpighian Tubules.

### rDNA localisation in mitotic and meiotic chromosomes

The results unequivocally indicated the existence of a single rDNA locus in mitotic chromosomes *Schwenkfeldina* (Fig. S7a) in contrast to what is usually observed in polytene chromosomes. Examination of a number of slides indicated that rRNA genes are localised at one end of the smallest mitotic pair. No evidence for DNA complementary to ribosomal probes other than a single terminal site was found in oocyte zygotene chromosomes of *Schwenkfeldina* sp. (Fig. S7b). All chromosome sets showed that the rDNA terminal locus is clearly visible in the sense that it has never been linked to the L chromosomes, namely those that are limited to germ lines.

### Proportion of rDNA and histone gene copy number in polytene and diploid cells

**Figure 7:**
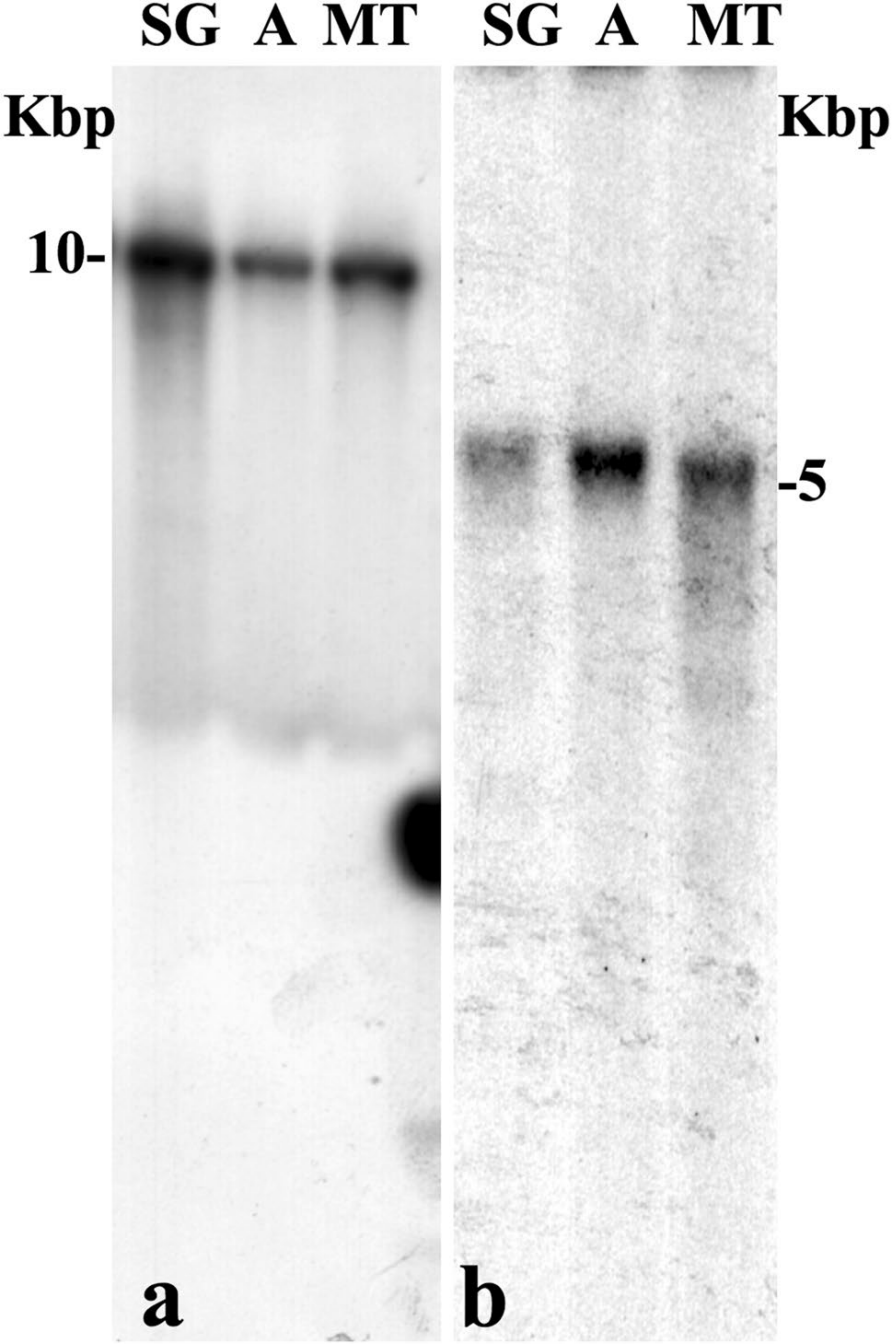
(**a**,**b**) *Schwenkfeldina* DNA from salivary gland (SG), adult flies (A) and Malpighian Tubules (TM) was cut with *Eco*RI, electrophorised in agarose gel, transferred to the nylon membrane and hybridised to rDNA (**a**) and histone gene (**b**) probes. Size markers in Kilobase pair (Kbp).

The nucleolar fragmentation visualised as rDNA dispersion in *Schwenkfeldina* sp. raised the question on DNA replication and its relationship with rDNA copy number in this species. Southern-blot hybridisations were then carried out not to evaluate its rDNA absolute copy number but rather to establish a relative comparison between different cell types. Possible variations could be detected by comparing the intensity of Southern-blot hybridisation signals, with particular regard to polytene tissues relative to those that do not undergo polyteny. Quantification and sample loading were performed with extreme care to ensure that equal DNA amount from distinct cells were loaded in gels. DNA from adults was chosen as mostly representing diploid cells to be compared to those from salivary gland and Malpighian Tubules (Fig. 7a). Results obtained in three experiments showed that salivary gland lanes always displayed higher densitometric values (1.4X ± 0.3) than those measured in adult lanes, the same occurring with the lane corresponding to Malpighian Tubules (1.2X ± 0.2). Histone gene repeats were introduced in the assay as a further reference for gene copy number in diploid and polytene cells of *Schwenkfeldina* sp. The choice is justified by general genomic features shared by ribosomal and histone gene repeats^25^ although the latter do not undergo dispersion as the rDNA of this species does. The *Eco*RI restriction site defines, as for the rDNA, the length of the histone repeat unit as a single band migrating with the expected size of 5 Kbp hybridised to the probe (Fig. 7b). Results from densitometry showed that the intensity of the histone probe signal was higher in the adult lanes (2.2X ± 0.2X) in three experiments performed.

### Chromosomal distribution of H3K9Me in *Schwenkfeldina*

**Figure 8:**
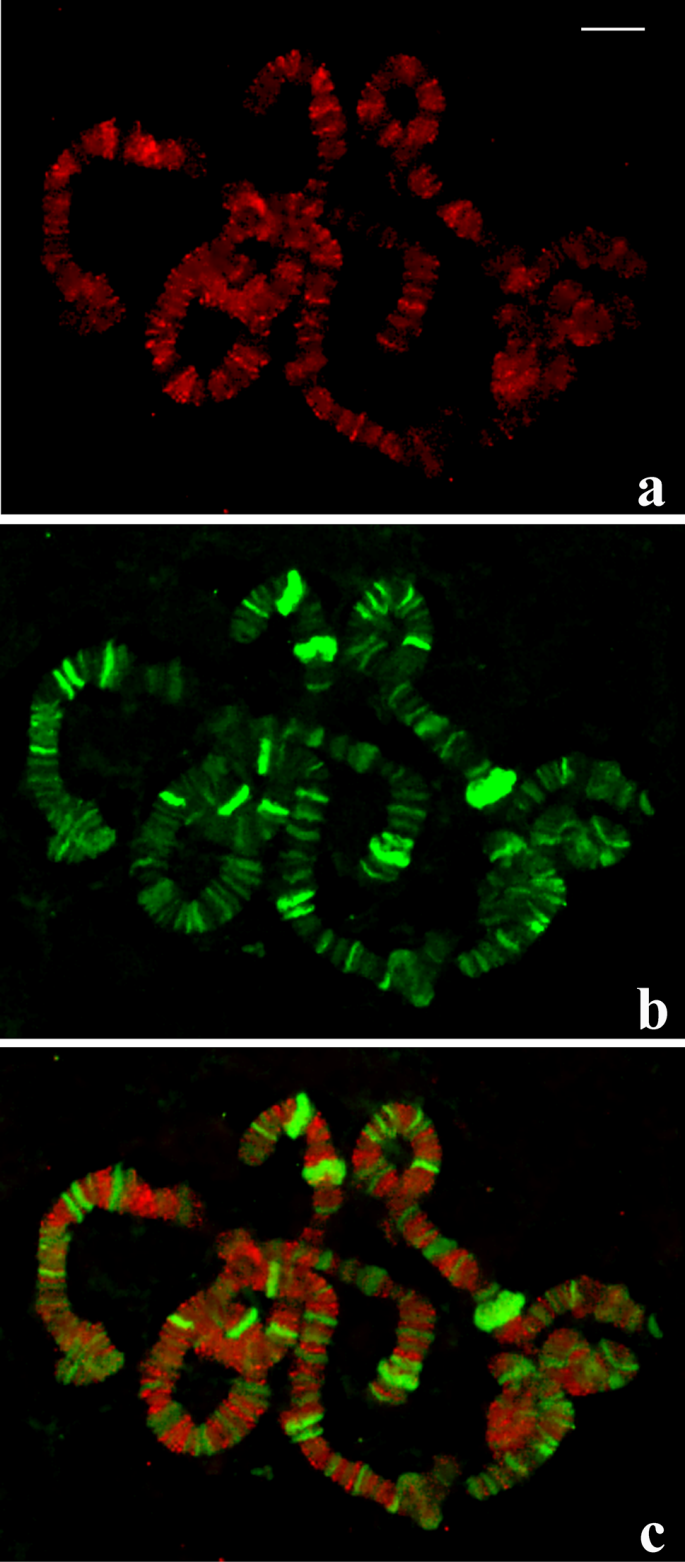
(**a–c**) Distribution of H3K9Me1-3 and RNA polymerase II in *Schwenkfeldina* sp. polytene chromosomes. Anti-H3K9Me1-3 labelling (**a**), anti-RNA polymerase II labelling (**b**) and the merged signals (**c**). Visualisation of signals from the two markers indicates that both do not usually overlap. Bar = 30 µm.

In polytene chromosomes of sciarid flies, heterochromatin has been identified morphologically by its condensed aspect and localisation in pericentric areas that sometimes lack the typical banding pattern of euchromatin. Heterochromatin may also appear in the form of chromosome breakpoints as a result of local DNA under-replication. As seen in *R. americana*, *T. pubescens* as well as in many organisms, heterochromatin is in addition characterised by the enrichment with methylation of histone H3 in lysine 9 (H3K9Me1-3)^26,27^. In *Schwenkfeldina* sp., rather than lack of banding pattern in condensed pericentric heterochromatin, its occurrence is immediately suggested by polytene chromosome breakpoints localised in several interstitial regions. Antibodies to the above mentioned epigenetic markers were employed to assess its distribution in polytene chromosomes. Experiments were done with either mixing antibodies to H3K9Me1-3 or with antibodies diluted individually; the results were the same in any case. A broad distribution of the heterochromatin markers was detected throughout the polytene chromosomes with several sections displaying brighter signals (Fig. S8). Controls were done using the same antibody dilutions in polytene chromosomes of *D. melanogaster* and showed the expected results as demonstrated previously (^28,29^, Fig. S9). Although a chromosome map of antibody binding has not been provided, the regions stained by the antibodies coincide with those from the rDNA attachment. The results cxobtained for the first time in this species led us to investigate the chromosomal distribution of H3K9Me1-3 and its relationship with transcriptional activity in the salivary gland. Double labelling using antibodies to H3K9Me1-3 and RNA polymerase II was then carried out. The results indicated that, although signals from the two markers overlap in some cases, most chromosome regions showed that the detection of H3K9Me1-3 and RNA polymerase II is mutually exclusive (Fig. 8).

### Chromosomal distribution of rDNA and H3K9Me in *R. americana* chromosomes

In a short report, the rDNA dispersion in the salivary gland of *R. americana* was demonstrated^18^. Such a process occurs regularly and results observed in polytene (Fig. S10) as well as in non-polytene chromosomes (Fig. S11) are readily reproducible in this species. In mitotic chromosomes, the NOR is located in the proximal end of the X chromosome; in zygotene chromosomes this region appeared invariably associated with L chromosomes (Fig. S11b) in contrast to the *Schwenkfeldina* sp. NOR (Fig. S7). Also, the polytene chromosome structure of *R. americana* is well defined with particular regard to the pericentric heterochromatin. These regions are distinguished by a significant enrichment with H3K9Me1-3 visualised as strong signals from the antibody binding (^26,27^; Fig. S12). Micronucleoli in *R. americana,* as seen in *Schwenkfeldina* sp., disperses preferentially to chromosomal regions significantly reactive to antibodies to H3K9Me1-3 (Fig. S13).

## Discussion

After collecting *Schwenkfeldina* for the first time, early cytological observations already indicated its unusual nucleolar structure in the salivary gland. In relation to data obtained in *S. coprophila*, *T. pubescens*, *Rhynchosciara* species, *Sciara pauciseta*^30^ and *Sciara ocellaris*^31^, *Schwenkfeldina* sp. represents the most extreme case of nucleolar/rDNA dispersion. The results presented in this work have been exhaustively reproduced in the laboratory with different larval groups of this species since the first in situ hybridisation experiments.

The localisation of the rDNA in polytene chromosomes of *Schwenkfeldina* sp. is due to the attachment of micronucleoli originating from a single locus, as observed after hybridisation results in mitotic chromosomes where the NOR is located at one end of the smallest chromosome pair which is not the X according to the sciarid chromosome nomenclature^2^. This question could be resolved in this work by performing in situ hybridisation in male mitotic chromosomes (sex-determination system X0) that have not been available. Unless this species has an exceptionally short X chromosome, the data imply the existence of sciarid species with rDNA loci in chromosome C rather than the sex chromosome. Support for such a possibility also comes from other sciarid, *Bradysia hygida*, whose NOR is localised at the pericentric end of chromosome C^32^.

The rDNA dispersed is contained in multiple ectopic nucleoli of *Schwenkfeldina* sp. Such micronucleolar regions could be called "extra-chromosomal loci" since integration of dispersed rDNA into the chromosome structure is unlikely. Support for the latter assumption comes from data indicating that a significant amount of rDNA is not integrated to the polytene chromosome structure of *S. coprophila*^33^. Since hybridisation occurred regularly to several chromosome regions of *Schwenkfeldina* sp. and also to chromosome sections C-11, B-15, X-1 and B-13 of *R. americana,* the "extra-chromosomal rDNA loci" cannot be explained as a random adherence of micronucleoli to these regions.

Among the processes that lead to nucleolar fragmentation and concomitant rDNA dispersion, DNA replication cycles for polytenisation seems to be one of these requirements. Support for this idea comes from the detection of rDNA dispersion restricted to nuclei undergoing polyteny. However, additional partners that are still unknown are involved in the control of this process, an assumption justified by the following examples. The extent of rDNA dispersion seemed to be a function of the number of DNA replication cycles (Figs. 3, 4); on the other hand, chromosomes displaying comparatively lower polyteny degree may present significant nucleolar fragmentation (Fig. 5). A further example showed rather a low degree of rDNA dispersion in nuclei that underwent low polytenisation (Fig. 6). The data suggest that nucleolar/rDNA dispersion in the salivary gland is differentially controlled depending on the cell type.

Southern-blot hybridisation data indicated that the proportion of rDNA in the two polytene tissues of *Schwenkfeldina* sp. is higher than in adults, the later assumed to represent diploid cells. These results were unexpected since in *R. americana* the rDNA was reported have approximately half of copy number for salivary gland in relation to the ovary^34,35^. As the reference for diploid tissues used in this work was adult flies rather than ovary, this might explain the discrepancy between the two reports. In contrast, histone genes are under-replicated in polytene tissues compared to adult cells in all the experiments done. Under-replication of sequences located in the heterochromatin is generally due to their delayed replication compared with the bulk of euchromatic DNA as early observed in *Drosophila*^36,37^. All the sciarid species that have been studied to date have NORs in the heterochromatin (see references in Introduction) so that rDNA under-replication is expected. Numeric values for the rDNA in polytene cells of *Schwenkfeldina* sp., albeit higher, are not very far from those measured for adults. For this reason, rDNA amplification, as historically detected in amphibian oocytes^38^, may not be the exact definition for this finding. In this sense, it is worth mentioning that dispersed nucleolar material in *Sciara* raised the hypothesis on the extra-chromosomal rDNA generation as a device to restore the rDNA content in salivary gland nuclei^33^. Multiple nucleoli in *Schwenkfeldina* sp. together with the lack of rDNA under-replication in polytene nuclei is likely to play a role in order to counterbalance the rDNA loss that occurs in polytene cells due to the chromatin under-replication as suggested by previous data and reinforced by the results obtained in this work.

The chromosomal location of H3K9Me1-3, unusual at a first glance, agreed with early cytological findings. Several interstitial breakpoints in chromosomes of *Schwenkfeldina* sp. suggested that heterochromatic regions were spread throughout the chromosome length. Also, the distribution of H3K9Me1-3, the most conserved epigenetic marker for the heterochromatin, relative to RNA polymerase II may be viewed as an expected result regarding, if any, low transcriptional activity at those sites where H3K9Me1-3 was detected. As the extra-chromosomal rDNA within micronucleoli is usually associated or close to the regions defined epigenetically as heterochromatin, it must play a role in order to establish the rDNA topology in sciarid polytene nuclei. Such an assumption is supported by *R. americana* data showing that the distribution of extra-chromosomal DNA follows the location of regions that are distinguished by the enrichment with H3K9Me1-3.

The nucleolar scenario in *Schwenkfeldina* sp. salivary gland resembles to some extent that reported for the salivary gland chromosomes of *Pseudodiamesa branickii* (Diptera: Chironomidae). Chromosome 4 of this species carries the NOR and DNA under-replication impedes its complete polyteny so that only three chromosomes are seen in salivary gland nuclei. Selective polytenisation leads to rDNA replication forming multiple nucleoli associated to many polytene regions of the three *Pseudodiamesa* polytene chromosomes^39^. The intriguing nucleolar picture in the chironomid salivary gland is morphologically reminiscent of nucleolar dispersion detected in *Schwenkfeldina* sp. Whether it implies either under-replication or amplification processes has remained speculative.

Hybridisation experiments have not been performed with the chromosomes of the sciarid *Hybosciara fragilis*, as it has not yet been re-collected. This species is likely to fall in the *Schwenkfeldina* group since nucleolar-like bodies in *Hybosciara* displayed a chromosomal distribution resembling that observed in *Schwenkfeldina*. The morphological aspect of those structures suggests that they are indeed nucleolar organisers attached to the chromosomes^10^. In summary, rDNA dispersion in *Schwenkfeldina* sp. probaly represents, rather than an exception, a dramatic example of a process that occurs in sciarid cells undergoing polyteny. Further functional implications from the preference of dispersed rDNA for the heterochromatin in *Schwenkfeldina*, *Rhynchosciara* and other members of Sciaridae cannot be explained at present and remains to be investigated.

## Methods

### Animals

*Schwenkfeldina* sp. adults were collected on the campus of the University of São Paulo (we thank Dr. D. S. Amorim for identification of the genus). Larvae were kept in the laboratory at 18 °C and food consisted of fresh *Sterculia chicha* flowers squashed in water. *Rhynchosciara americana* larval groups were collected in private banana plantations in the region of Mongaguá, São Paulo. Larvae were kept in the laboratory at 18 °C and food consisted of fresh potato leaves. *Drosophila melanogaster* wild type (Oregon R) came from laboratory stocks.

### Preparation of chromosome and embryonic nuclei spreads

Salivary glands and Malpighian Tubules were dissected in 1X PBS and fixed briefly in ethanol-acetic acid (3:1). After squashing the glands in 50% acetic acid, the slides were frozen in liquid nitrogen. The coverslips were flipped off with a razor blade and the slides were then kept in absolute ethanol at − 20 °C. Preparation of chromosome spreads from dissected brain ganglia and ovarian follicle cells was carried out as described^40^ and the slides stored as above. Embryos were immersed in 2.5% sodium hypochlorite for 5 min, washed in 1X PBS, 0.1% Tween 20. After keeping the embryos in heptane for 10 min, fixation was made with 70% ethanol and the squashes were done in 50% acetic acid following additional procedures as described above. For chromosomal protein detection, salivary glands were squashed in a solution containing 3.7% formaldehyde, 40% acetic acid following the same procedures as described above.

### Preparation of the probes

A sub-clone containing the complete rDNA repeat unit of *R. americana* named p*Ra*-1.4^41^ was used as rDNA probe. Plasmid DNA extraction was done according to current protocols (Life Tech) and labelled by nick translation with either biotin or digoxigenin following the instruction of the manufacturer (Roche). An alternative RNA probe was prepared with insect total RNA extraction (TRIzol, Life Tech) dissolved in formamide containing an excess of poly (r)-U (Roche). Total RNA is predominantly composed of rRNA but messenger poly-(r)A tails are present and are apt to hybridise to poly (d)A.(d)T-rich chromosomal regions^24^. This has to be avoided, particularly for *Rhynchosciara* species, if the detection is carried out with the anti-RNA.DNA hybrid antibody. Poly (r)-U in the hybridisation mixture will form complexes with poly (r)-A tails, preventing them from hybridising to homopolymer-rich chromosomal DNA^42^. Use of the rRNA probe made as described above reproduced the same hybridisation results observed with rDNA probes.

### In situ hybridisation

When the RNA probe was used, the air-dried chromosome squashes were treated with RNase A (0.6 mg/ml) diluted in 2X SSC at 37 °C for at least 2 h in a moist chamber. The probe mixtures consisted of 50% formamide, 2X SSPE, 0.1% SDS, 30 ng/µl labelled DNA or 200 ng/µl insect RNA mixed with poly (r)-U. Six µl of probe mixture was applied to each air-dried slide and covered with a plastic coverslip. Metaphase and polytene chromosome slides were steam heated at 75 °C for 10 min to denature both chromosomal DNA and probe, and then kept in a closed box at 37 °C overnight (up to 48 h for metaphase chromosomes) for hybridisation. When DNA probes were used, chromosome squashes were treated with RNase A (0.6 mg/ml) diluted in 2X SSC at 37 °C for at least 2 h in a moist chamber. Hybridization procedures and detection of DNA labelled probes were described elsewhere^17–20^. When the RNA probe was used, the slides were washed twice in 0.1X SSPE, 0.1% SDS at 37 °C, followed by incubation at room temperature in 1X TBS, 0.1% Triton X-100 (TBST), 10% Superblock (Pierce) for 30 min. The slides were incubated with goat IgG anti-RNA.DNA hybrid^22^ diluted 1:50 in TBST/Superblock solution. After 1 h at room temperature in a moistened chamber, the slides were washed twice in TBST for 30 min and then incubated with rabbit IgG anti-goat labelled with TRITC (Sigma) diluted 1:200 as described above. The slides were washed twice in TBST for 30 min and finally once in 1X TBS for 5 min. Chromosomes were stained with 4′, 6-diamidino-2-phenylindole (DAPI). The slides were mounted in antifading medium (Vectashield, Vector Labs) and inspected with epifluorescence optics (Nikon).

### Endogenous hybridisation

Formation of endogenous RNA.DNA hybrids was performed by fixing salivary glands in ethanol-acetic acid (3:1) and squashing in 50% acetic acid, following the same steps described for preparation of chromosome spreads. The slides that were kept frozen in ethanol were hydrated in 1X TBS and subsequently incubated in 50% acetic acid at 42 °C for 2–5 min. Detection of endogenous RNA.DNA hybrid, control with RNases and mounting of the slides were done as described for in situ hybridisation with RNA probe.

### Immunolabelling

The slides were rehydrated in 1X TBS followed by incubation at room temperature in TBST, 10% Superblock (Pierce) for 20 min. Monoclonal antibodies to RNA polymerase II (Upstate Tech, clone CTD4H8) as well as polyclonal antibodies to the three methylation forms of histone H3 in lysine 9 (Upstate Tech 07-440, 07-441, 07-442) were diluted 1:100 in TBST/Superblock solution. The incubations were done in a moistened chamber at room temperature for 2 h. After washes in TBST, the slides were incubated for 1 h at room temperature with a mixture containing goat IgG anti-rabbit IgG conjugated with TRITC (Sigma) and goat IgG anti-mouse conjugated with Alexa Fluor 488 (Molecular Probes), both diluted 1:200 in TBST. The slides were washed twice in TBST for 30 min and finally in 1X TBS for 5 min. Chromosomes were stained with 4′, 6-diamidino-2-phenylindole (DAPI). The slides were mounted in antifading medium (Vectashield, Vector Labs) and inspected with epifluorescence optics (Nikon).

### Southern blot hybridisation

DNA extraction was carried out according to standard protocols^43^. DNA samples (5–7 µg of each sample per lane) were quantified by spectrophotometry (NanoDrop ND-100, Uniscience) three times prior to digestion with *Eco*RI. Restriction enzymes and DNA size markers were purchased from New England Biolabs. DNA from gels was alkaline transferred to Hybond N + membranes according to standard procedures (GE Healthcare). Three hybridisation assays were carried out overnight at 60 °C in 0.5 M Na_2_HPO_4_, 2% SDS. Ribosomal DNA^41^ and histone gene^44^ inserts were labelled by random priming with ^⍺32^P-dATP following current protocols (Life Tech). The membranes were washed twice at 60 °C for 30 min in 40 mM Na_2_HPO_4_, 2% SDS. Signal quantification was performed with ImageJ 1.48 V Analysis Software (Wayne Rasband, NIH, USA).

## Supplementary Information

Supplementary Figure S1.

Supplementary Figure S2.

Supplementary Figure S3.

Supplementary Figure S4.

Supplementary Figure S5.

Supplementary Figure S6.

Supplementary Figure S7.

Supplementary Figure S8.

Supplementary Figure S9.

Supplementary Figure S10.

Supplementary Figure S11.

Supplementary Figure S12.

Supplementary Figure S13.

Supplementary Figure Legends.
