# Supplementary figures and images for: Dramatic nucleolar dispersion in the salivary gland of Schwenkfeldina sp. (Diptera: Sciaridae)
Source: Sci Rep. 2021 Apr 16;11:8347. doi: 10.1038/s41598-021-87012-5 (PMC8052372; doi:10.1038/s41598-021-87012-5)

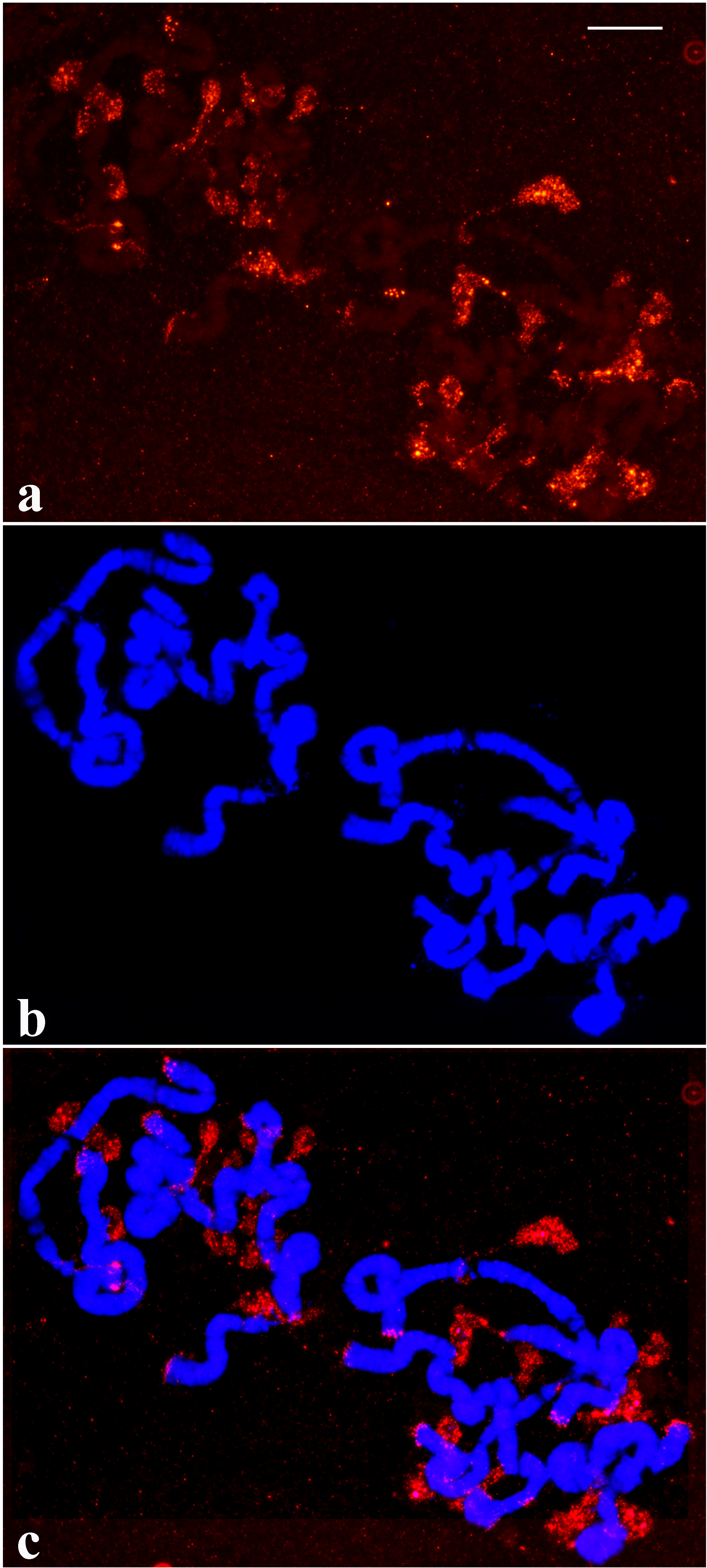

Supplement: Supplementary file 2 — Supplementary Figure S2. [file 41598_2021_87012_MOESM2_ESM.tif]

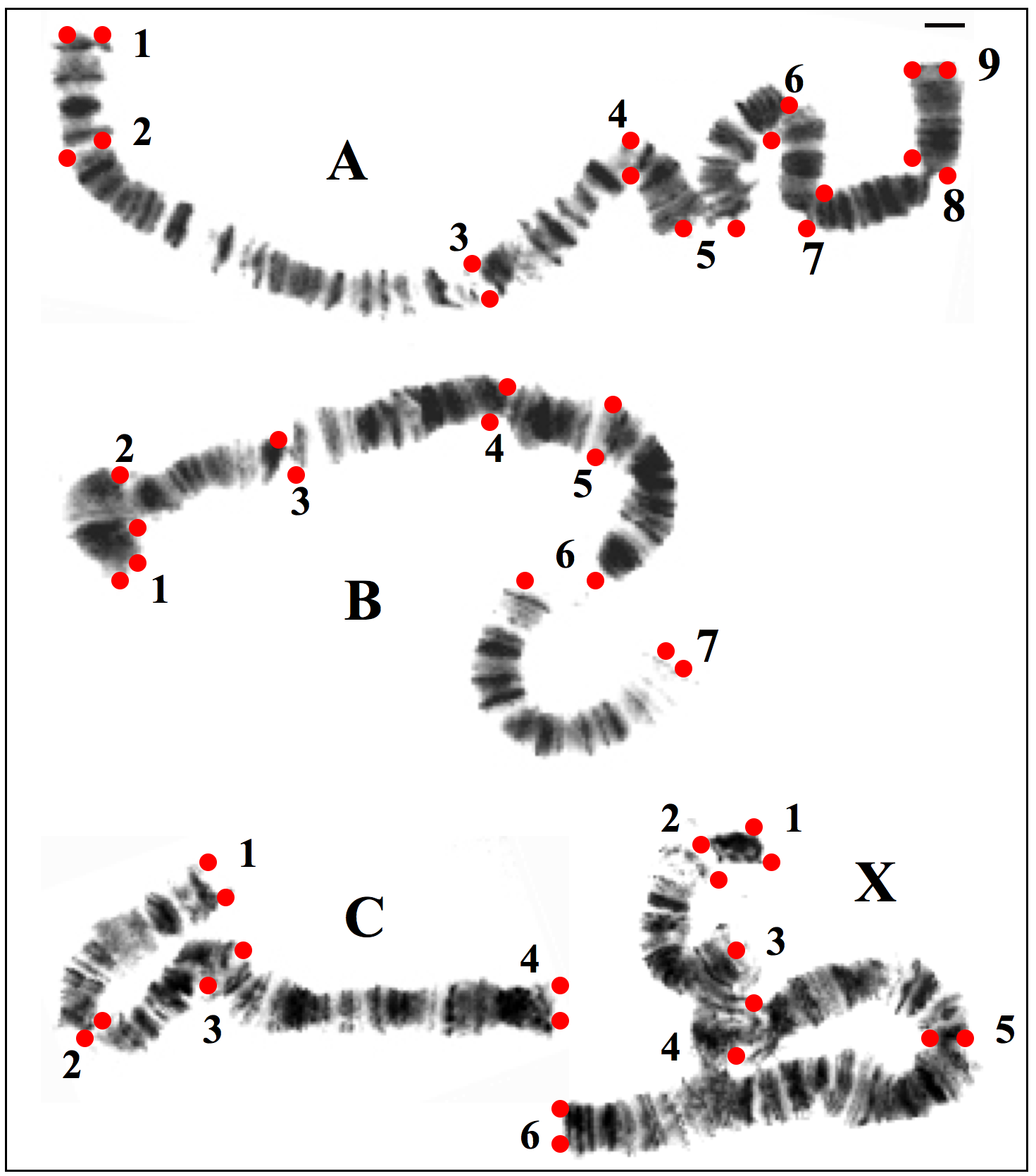

Supplement: Supplementary file 3 — Supplementary Figure S3. [file 41598_2021_87012_MOESM3_ESM.tif]

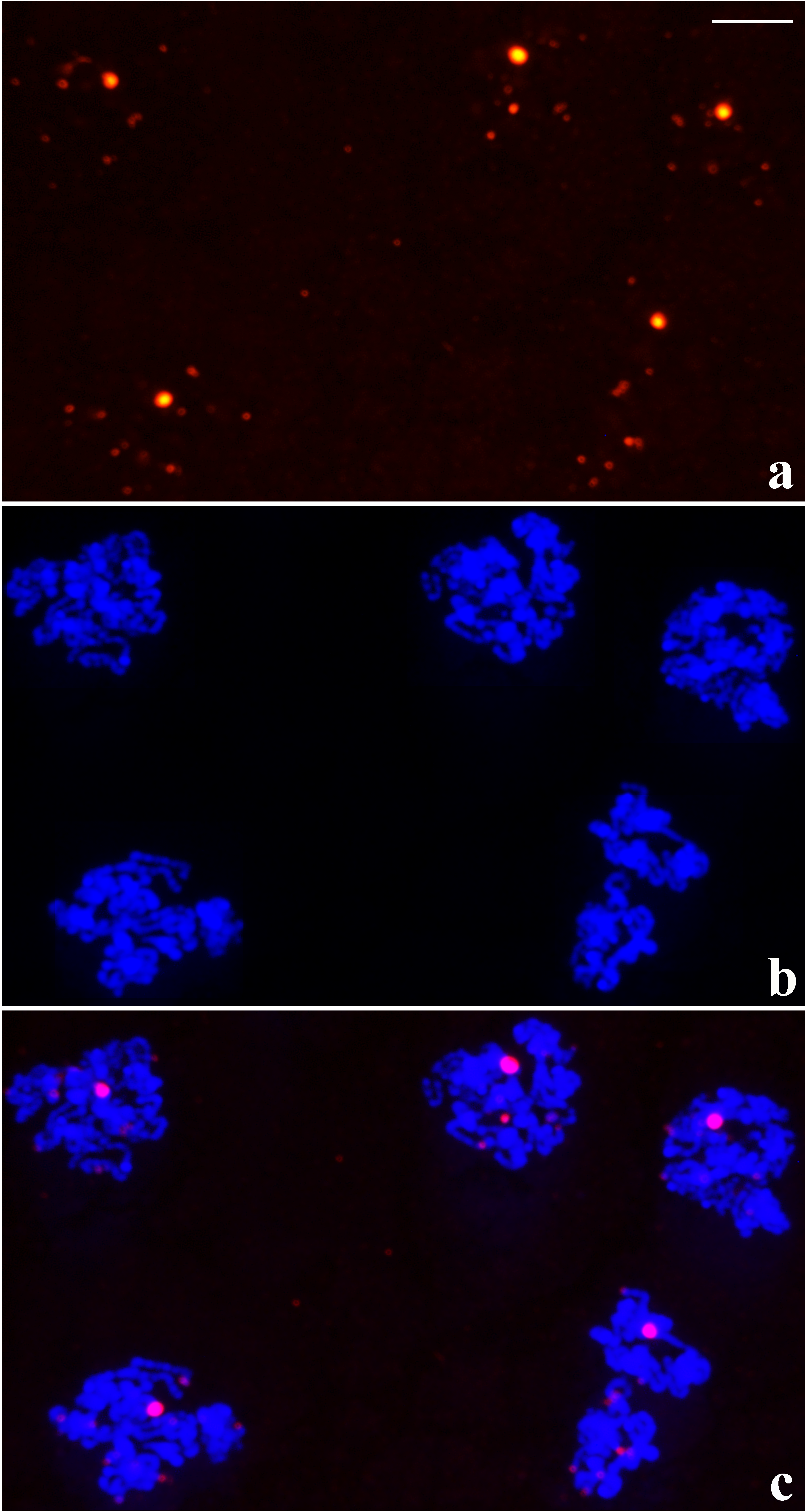

Supplement: Supplementary file 4 — Supplementary Figure S4. [file 41598_2021_87012_MOESM4_ESM.tif]

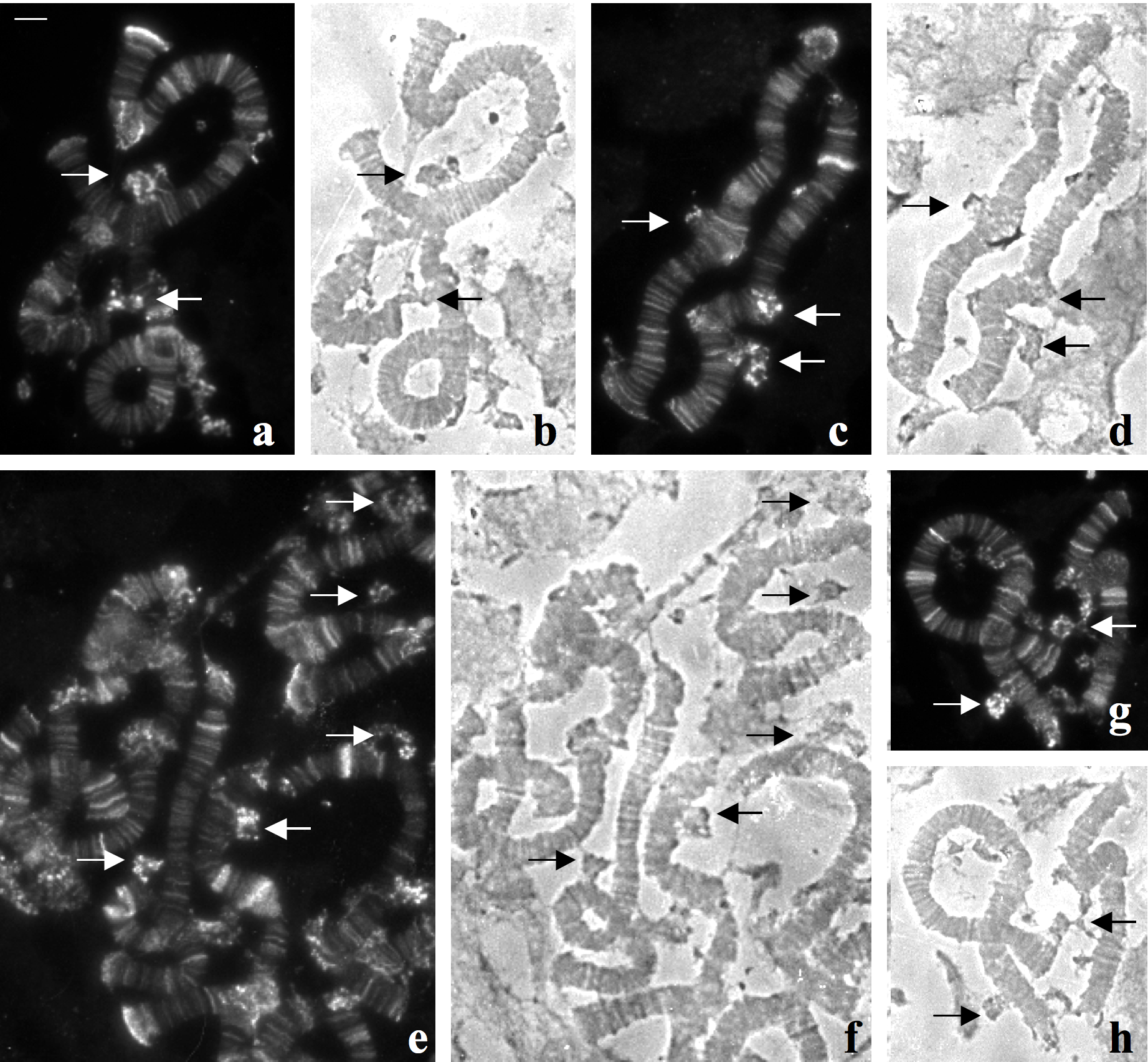

Supplement: Supplementary file 5 — Supplementary Figure S5. [file 41598_2021_87012_MOESM5_ESM.tif]

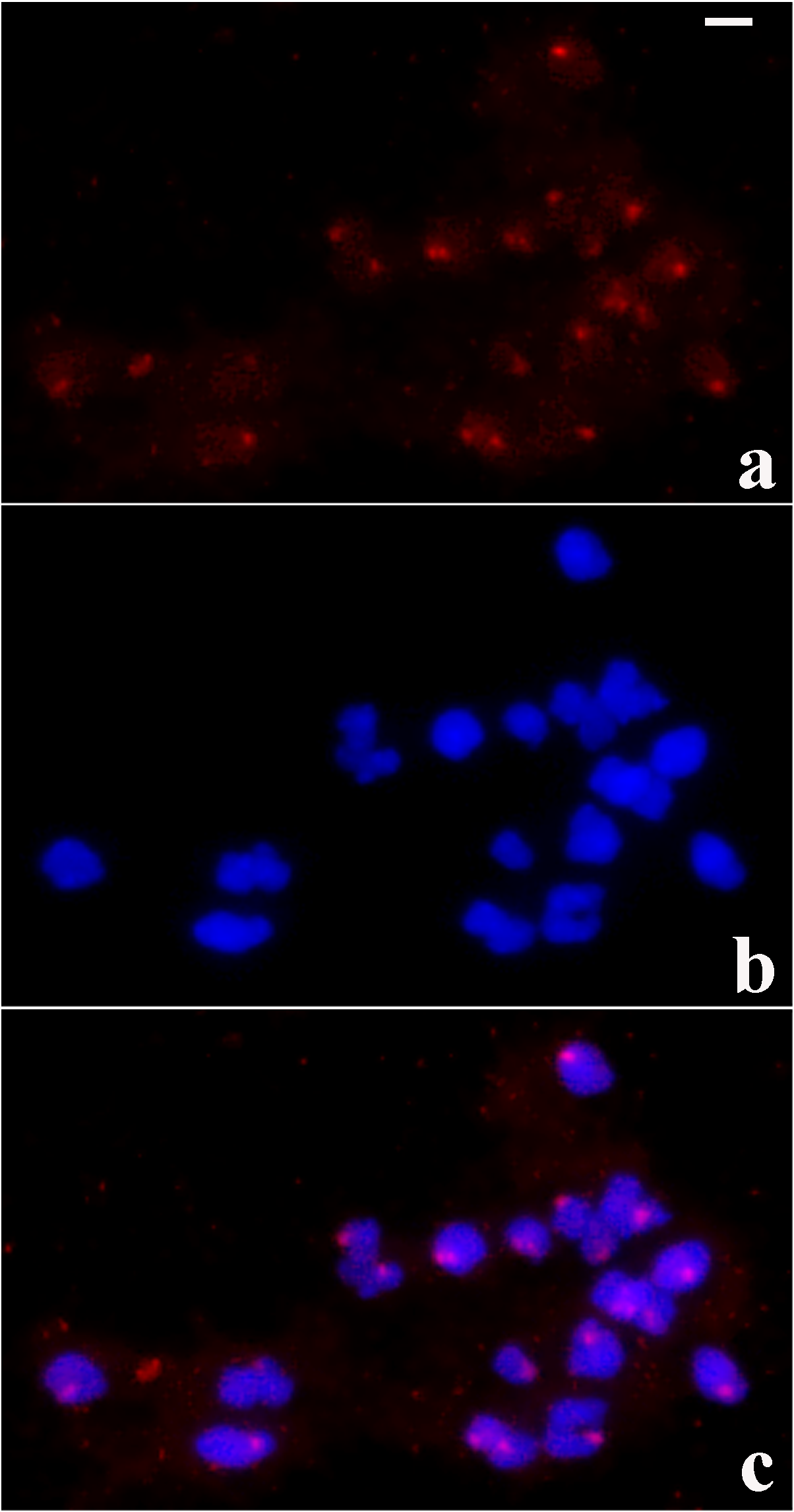

Supplement: Supplementary file 6 — Supplementary Figure S6. [file 41598_2021_87012_MOESM6_ESM.tif]

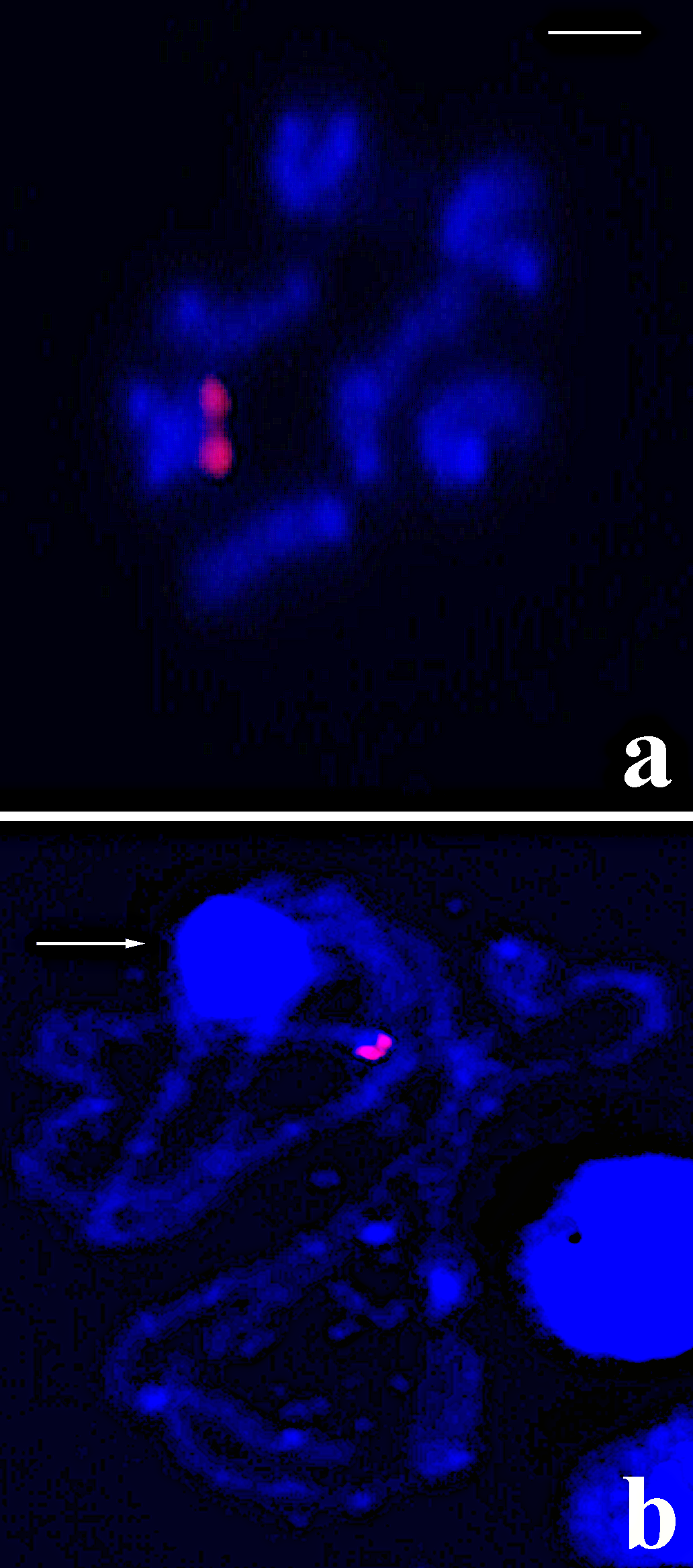

Supplement: Supplementary file 7 — Supplementary Figure S7. [file 41598_2021_87012_MOESM7_ESM.tif]

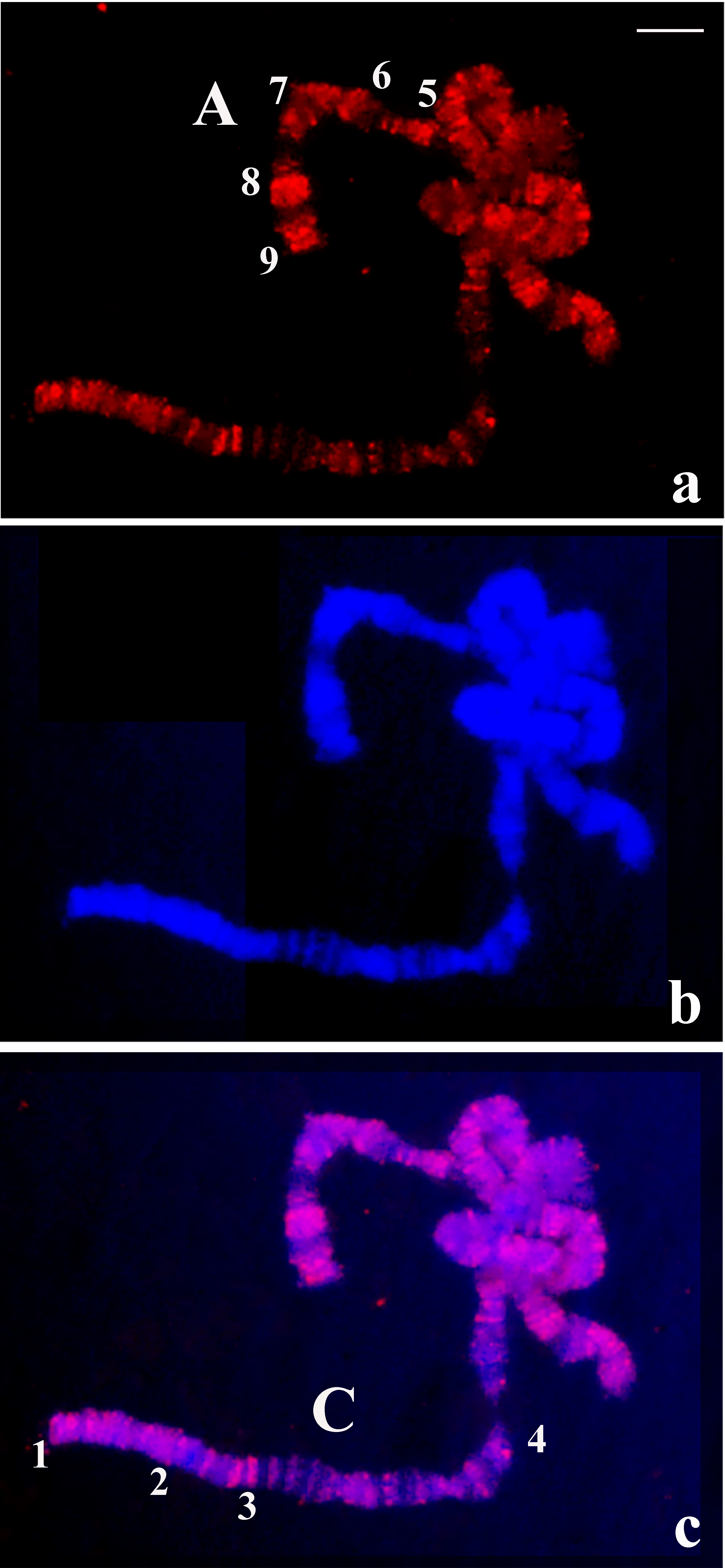

Supplement: Supplementary file 8 — Supplementary Figure S8. [file 41598_2021_87012_MOESM8_ESM.tif]

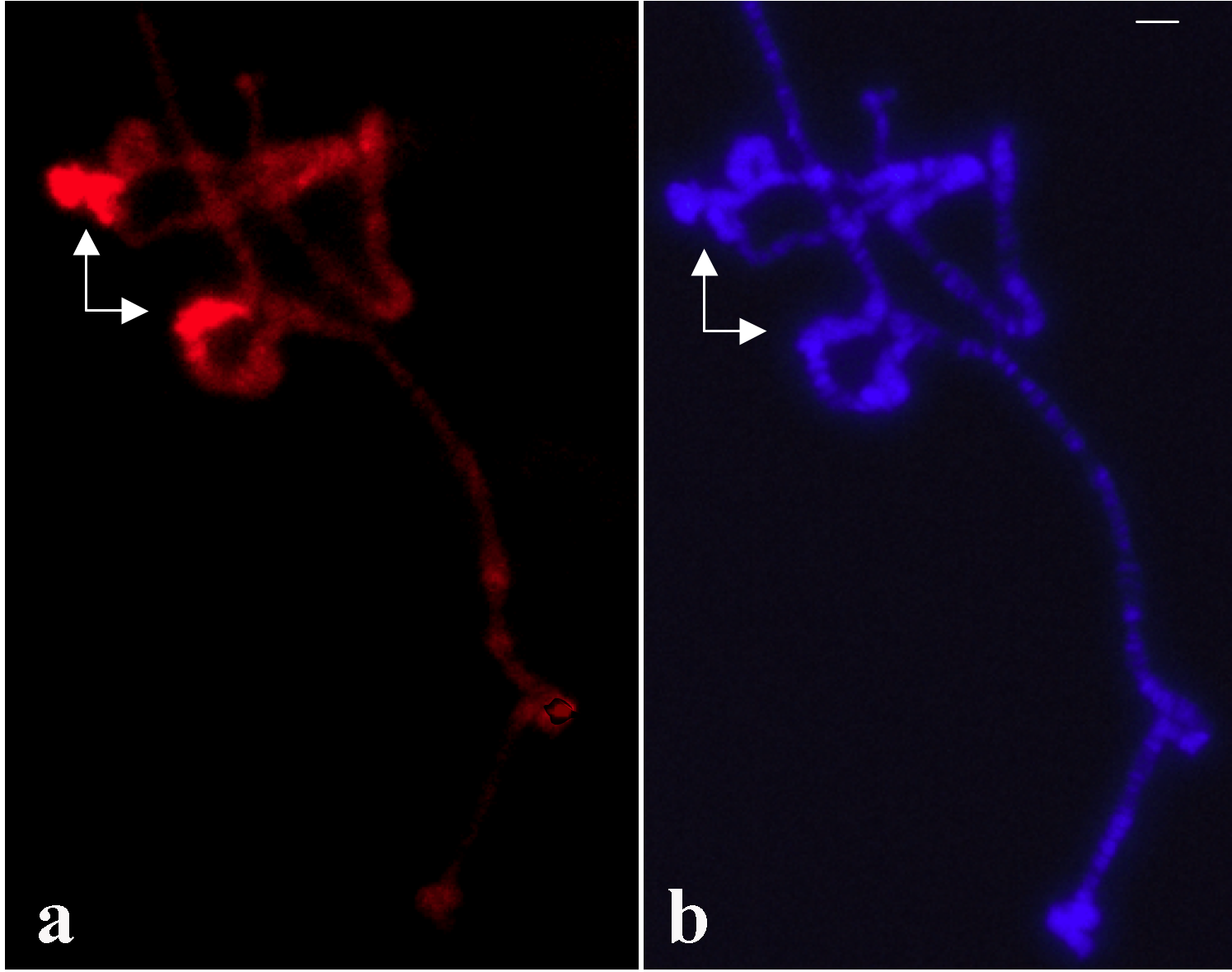

Supplement: Supplementary file 9 — Supplementary Figure S9. [file 41598_2021_87012_MOESM9_ESM.tif]

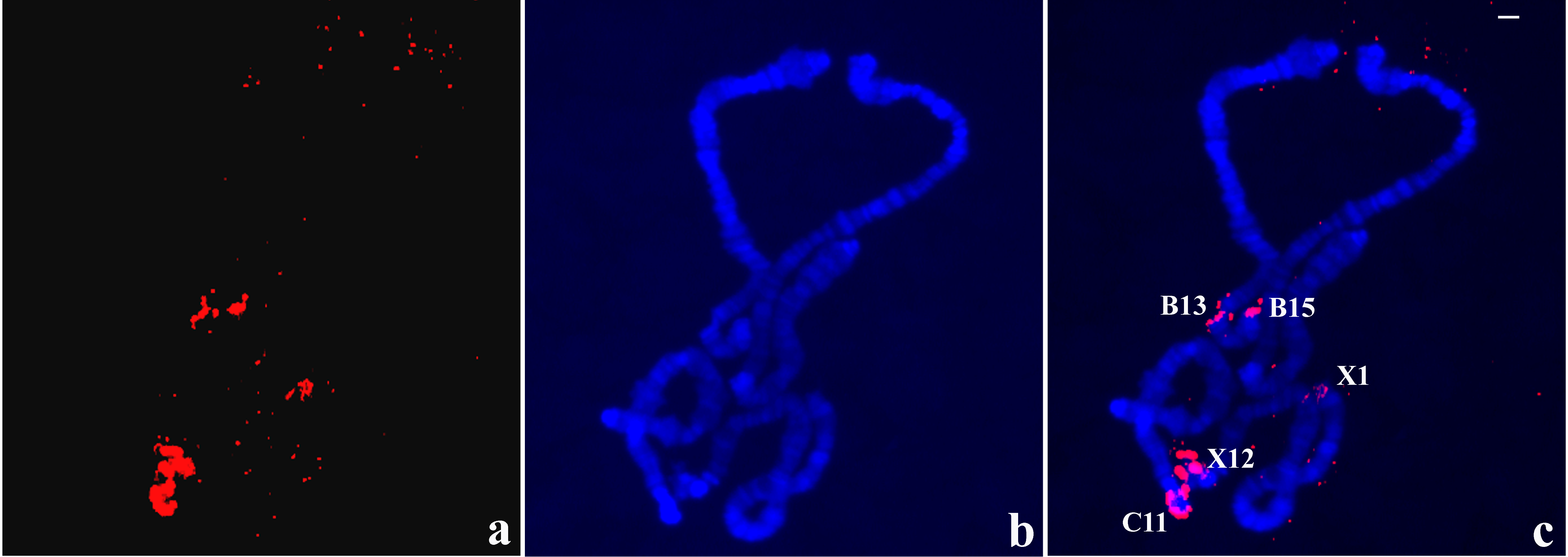

Supplement: Supplementary file 10 — Supplementary Figure S10. [file 41598_2021_87012_MOESM10_ESM.tif]

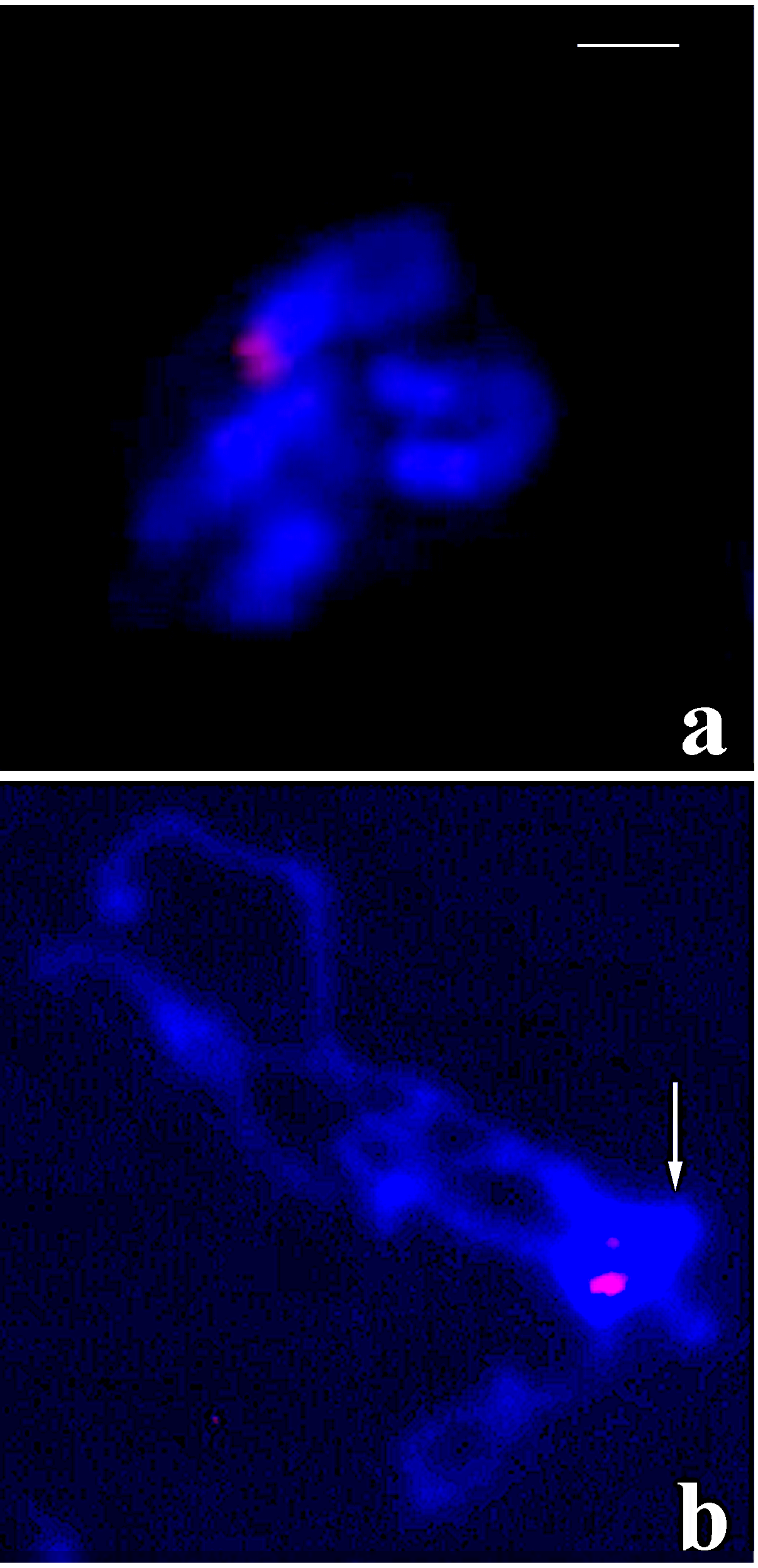

Supplement: Supplementary file 11 — Supplementary Figure S11. [file 41598_2021_87012_MOESM11_ESM.tif]

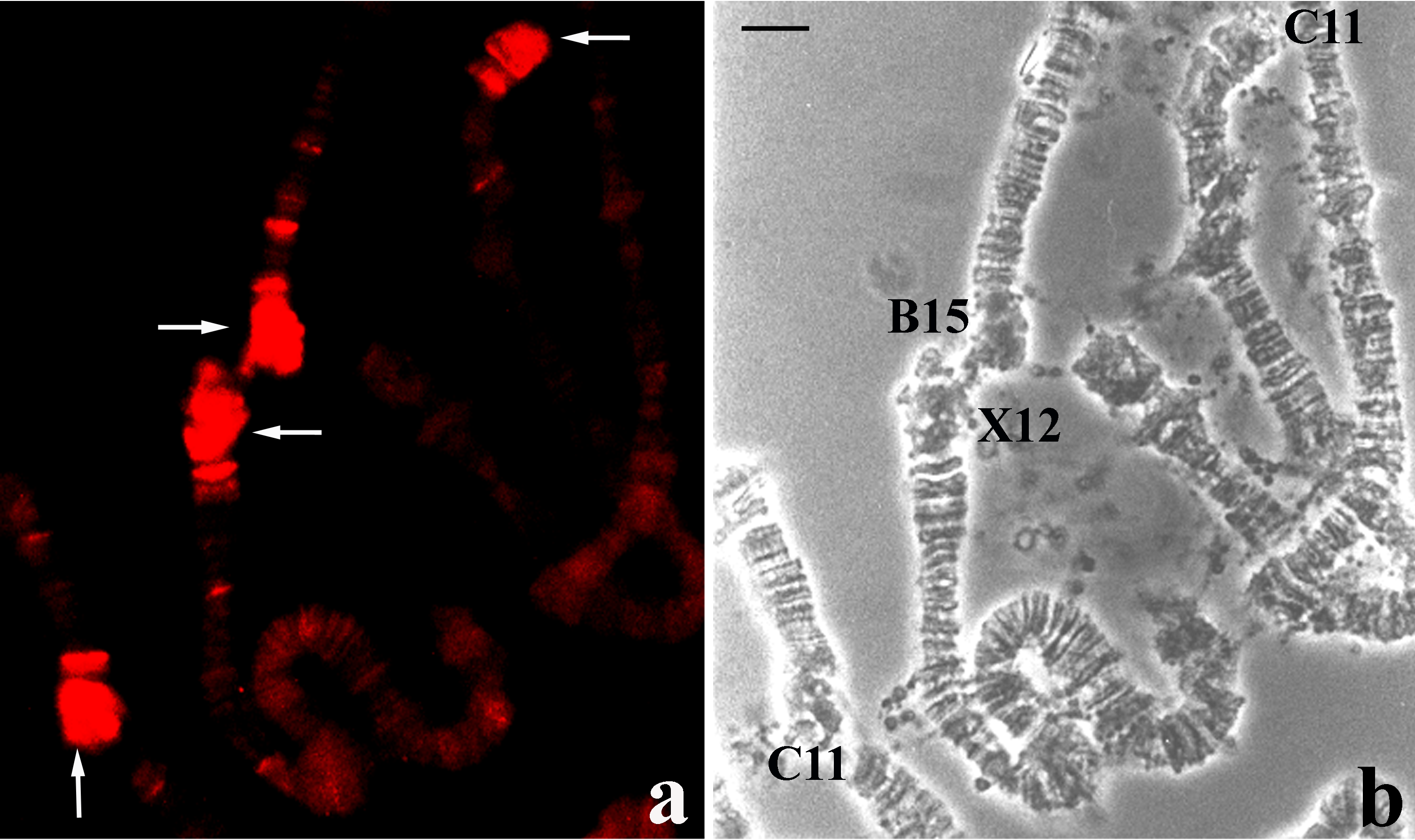

Supplement: Supplementary file 12 — Supplementary Figure S12. [file 41598_2021_87012_MOESM12_ESM.tif]

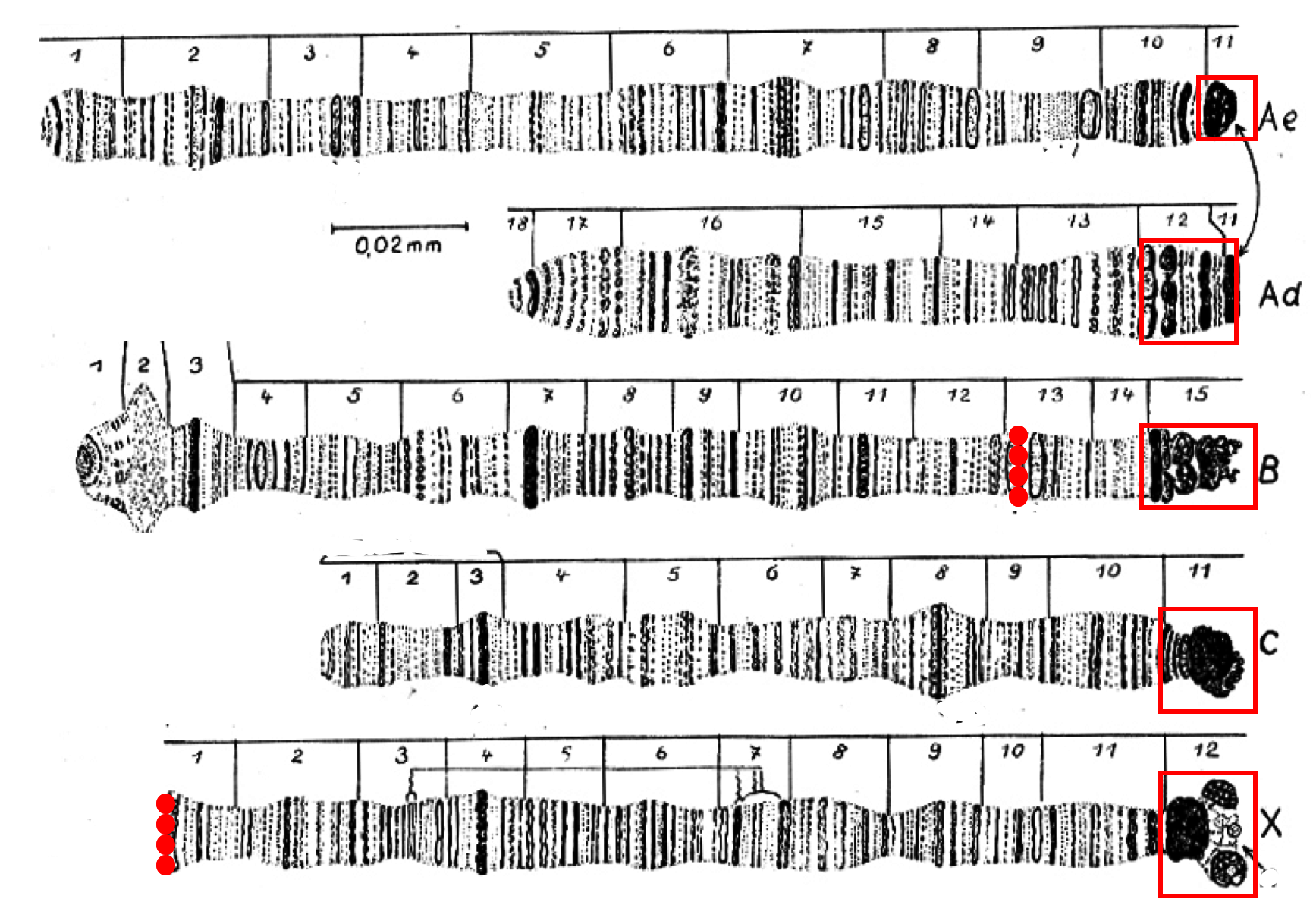

Supplement: Supplementary file 13 — Supplementary Figure S13. [file 41598_2021_87012_MOESM13_ESM.tif]
