# Supplementary material for: Dramatic nucleolar dispersion in the salivary gland of Schwenkfeldina sp. (Diptera: Sciaridae)
Source: Sci Rep. 2021 Apr 16;11:8347. doi: 10.1038/s41598-021-87012-5 (PMC8052372; doi:10.1038/s41598-021-87012-5)
Supplement: Supplementary file 14 — Supplementary Figure Legends. [file 41598_2021_87012_MOESM14_ESM.docx]

**Fig. S1** Polytene chromosomes of *Schwenkfeldina sp*. The arrows point to some examples of structures, either disconnected or attached to the chromosomes, that were eventually identified as micronuclei on the basis of hybridisation results. Six regions, identified in the figure (**1-6**), from distinct chromosomes that were identified as B chromosomes according to previous criteria [1] were found to be more frequently associated with micronucleolar bodies. Bar =15 µm

**Fig. S2** Localisation of the rDNA in *Schwenkfeldina sp.* polytene chromosomes using RNA probe. Fluorescent hybridisation signals (**a**) appear in larger areas in relation to Fig. 3; DAPI staining (**b**) and merged signals (**c**). Bar = 45 µm

**Fig. S3** Polytene chromosomes of *Schwenkfeldina sp.* were identified (**A**, **B**, **C**, **X**) according to previous criteria [1]. Numbering and labelling (**red**) indicate the most frequent sites of rDNA localisation, using either RNA or DNA probes. Identification of these sited was made using data from seven slides and 43 chromosomes of each type. Bar = 15 µm

**Fig. S4** Localisation of the rDNA in *Schwenkfeldina sp.* polytene chromosomes from posterior cells of the salivary gland showing RNA probe signals (**a**), its corresponding DAPI staining image (**b**) and the merged signals (**c**). The images provide visualisation of a main rDNA site in all the polytene nuclei as inferred by the intensity of its fluorescent area. Bar = 80 µm

**Fig. S5 a-h** Detection of endogenous RNA.DNA hybrids in *Schwenkfeldina sp*. polytene chromosomes. Fluorescent antibody signals (**a, c, e, g**) and the corresponding phase contrast images (**b, d, f, h**). The arrows point to some examples in which the hybrid detection is seen in micronucleolar bodies. Bar = 15 µm

**Fig. S6 a-c** Localisation of the rDNA in *Schwenkfeldina sp.* early embryonic nuclei showing RNA probe signals (**a**), the corresponding DAPI staining image (**b**) and the merged signals (**c**). A single rDNA signal is usually detected in embryonic nuclei. Bar = 5 µm

**Fig. S7 a,b** Localisation of the rDNA (red signal) in *Schwenkfeldina sp.* chromosomes stained with DAPI using RNA probe. Female metaphase showing signals in the smallest mitotic pair (**a**) and labelling at the single tip of the zygotene chromosome (**b**). The *arrow* points to the germ line-limited chromosomes devoid of rDNA signals. Bar = 15 µm

**Fig. S8 a-c** Chromosomal distribution of H3K9Me1-3 in *Schwenkfeldina sp.*. Antibody labelling (**a**), chromosomes stained with DAPI (**b**) and the merged signals (**c**). Numbering refers to preferential sites of micronucleolar attachment in chromosomes A and C according to data summarized in Fig. S3. Bar = 35 µm

**Fig. S9 a,b** Chromosomal distribution of H3K9Me1-3 in *Drosophila melanogaster*. Antibody labelling (**a**), chromosomes stained with DAPI (**b**). The *arrows* point to chromosome regions comprising pericentric and centromeric heterochromatin that are preferentially labelled by the antibodies. Bar = 10 µm

**Fig. S10 a-c** Localisation of the rDNA in polytene chromosomes of *R.* *americana.* DNA probe signals (**a**), the corresponding image stained with DAPI (**b)** and the merged signals (**c**). Chromosome sections (*B13, B15, C11, X1, X12*) were identified previously [45, 17]. Bar = 20 µm

**Fig. S11 a,b** Localisation of the rDNA (red signal) in *R. americana.* chromosomes stained with DAPI. Female metaphase showing signals only in the X mitotic pair (**a**) and the corresponding labelling as seen in zygotene chromosomes (**b**). The *arrow* points to the germ line-limited chromosomes that usually display rDNA signals in this species. Bar = 15 µm

**Fig. S12a,b** Chromosomal distribution of H3K9me3 in *R. americana***.** Indirect immunofluorescence (**a**) and the corresponding phase contrast image (**b**). Pericentric regions of polytene chromosomes (*B15*, *C11* and *X12*) were identified previously [45, 17]. Bar = 35 µm

**Fig. S13** Polytene chromosome maps of *R. americana* [45]. Labelling (**red**) in chromosome sections B13 and X1 indicate sites of rDNA and H3K9Me1-3 detection. Rectangles (**red**) point to the limits of pericentric regions of the four chromosomes where rDNA in addition to significant signals of H3K9Me1-3 were detected
